# Guest-Adaptable and Water-Stable Peptide-Based Porous Materials by Imidazolate Side Chain Control**

**DOI:** 10.1002/anie.201307074

**Published:** 2013-12-02

**Authors:** Alexandros P Katsoulidis, Kyo Sung Park, Dmytro Antypov, Carlos Martí-Gastaldo, Gary J Miller, John E Warren, Craig M Robertson, Frédéric Blanc, George R Darling, Neil G Berry, John A Purton, Dave J Adams, Matthew J Rosseinsky

**Affiliations:** Department of Chemistry, University of LiverpoolCrown Street, Liverpool, L69 7ZD (UK); Daresbury Laboratory, Science & Technologies Facilities CouncilKerkwick Lane, Warrington, WA4 4AD (UK)

**Keywords:** imidazolates, metal–organic frameworks, microporous materials, peptides, structural adaptability

## Abstract

The peptide-based porous 3D framework, ZnCar, has been synthesized from Zn^2+^ and the natural dipeptide carnosine (β-alanyl-L-histidine). Unlike previous extended peptide networks, the imidazole side chain of the histidine residue is deprotonated to afford Zn–imidazolate chains, with bonding similar to the zeolitic imidazolate framework (ZIF) family of porous materials. ZnCar exhibits permanent microporosity with a surface area of 448 m^2^ g^−1^, and its pores are 1D channels with 5 Å openings and a characteristic chiral shape. This compound is chemically stable in organic solvents and water. Single-crystal X-ray diffraction (XRD) showed that the ZnCar framework adapts to MeOH and H_2_O guests because of the torsional flexibility of the main His-β-Ala chain, while retaining the rigidity conferred by the Zn–imidazolate chains. The conformation adopted by carnosine is driven by the H bonds formed both to other dipeptides and to the guests, permitting the observed structural transformations.

Metal–organic frameworks (MOFs) are crystalline porous materials composed of inorganic nodes, either single ions or clusters of ions, bridged by organic linkers through metal–ligand coordination bonds.[1] Recently, several biomolecules, such as amino acids,[2] nucleobases,[3] saccharides,[4] and peptides,[5] were used as organic linkers in MOF synthesis, mainly because of the diversity of their metal binding sites. The incorporation of biomolecules in MOFs also attracts particular attention because they can improve the biocompatibility of the final products, enhance the structural and chemical diversity of the internal surfaces of MOFs, and afford chiral frameworks that may have unique separation and catalytic properties.[6]

Peptides are particularly interesting as linkers because dipeptides with hydrophobic residues that are held together by H bonds form metal-free purely peptide-based porous materials. These structures are divided into two groups, the Val-Ala compounds with hydrophobic pores and the Phe-Phe compounds with hydrophilic pores.[7] The Val-Ala structures exhibit typical CO_2_ and CH_4_ adsorption for microporous materials.[8]

In MOFs, peptides have the ability to act as connecting ligands as they have at least one amino and one carboxylic acid terminus that can coordinate metal ions. The dipeptides Gly-Ala and Gly-Thr thus connect Zn^2+^ ions to form two topologically distinct 2D-layered framework compounds, Zn(Gly-Ala)_2_ and Zn(Gly-Thr)_2_, respectively.[9] The former is a flexible porous material that displays an adaptable pore conformation, which evolves continuously from an open to a partially disordered closed structure in response to the level of guest loading. The latter is structurally rigid to guest loss in a manner characteristic of rigid MOFs and exhibits permanent porosity with a surface area of 200 m^2^ g^−1^ after solvent removal, as the framework is stabilized by the additional H bonding between the OH functional group from the threonine side chain and the NH_2_ terminal group. These two examples clearly show how diverse the structures can be and the strong control of adsorption behavior arising from small changes in the peptide unit.

Here we present a new peptide-based MOF, ZnCar⋅DMF, which is assembled from Zn^2+^ and carnosine (Car), a natural dipeptide with the molecular structure β-alanyl-l-histidine. The peptidic chain of carnosine contains an extra CH_2_ group compared to classic dipeptides, because of the β-amino acid structure of β-alanine. The histidine residue incorporates the imidazole moiety that serves as an additional metal binding site, and thus carnosine has two more potential linking points compared to Gly-Ala and Gly-Thr. In reported histidine-containing framework compounds the imidazole ring is neutral and binds only one metal atom at N3.[10] ZnCar⋅DMF is a 3D framework compound in which each carnosine molecule links four tetrahedral Zn cations, two of which are bridged by the deprotonated imidazolate ring. The structure is flexible and displays 1D permanent porosity upon removal of DMF. It is a microporous material with a specific surface area of 448 m^2^ g^−1^ and exhibits strong binding affinity for small molecules, such as CO_2_ and CH_4_. This MOF is not dissolved or decomposed in either H_2_O or MeOH. Moreover, after resolvation in MeOH and H_2_O, it adopts structures different from that of the as-made or desolvated material. Single-crystal X-ray diffraction studies showed that this flexibility is driven by H bonds formed between the guests and the dipeptide, leading to different torsional conformations of carnosine.

Crystals of ZnCar⋅DMF in the shape of rectangular prisms and 50 to 100 μm in length (Supporting Information, Figure S1) were obtained from the reaction of zinc nitrate with carnosine in DMF/H_2_O at 100 °C for 12 hours (see the Experimental Section in the Supporting Information). Under these conditions, both the imidazole N1 and the carboxy group of the carnosine are deprotonated to form the Car^2−^ anion, allowing the formation of a 4:4 motif with Zn^2+^ (Scheme 1). The Zn cations are tetrahedrally coordinated to four carnosine ligands (Scheme 1), and each ligand is coordinated to four Zn^2+^ ions by the C-terminal His carboxylate group, the N-terminal Ala amine group, and the two nitrogen atoms of the imidazole ring (Scheme 1). The chemistry and the bridging connectivity between the side chain imidazole of histidine and the Zn cations are reminiscent of those seen in ZIF compounds.[11] The molecular cyclic tetramer [Au(gly-L-his^3−^)]_4_⋅10 H_2_O is the only other known compound of histidine where metal–imidazolate bonding is employed.[12]

**Scheme 1:**
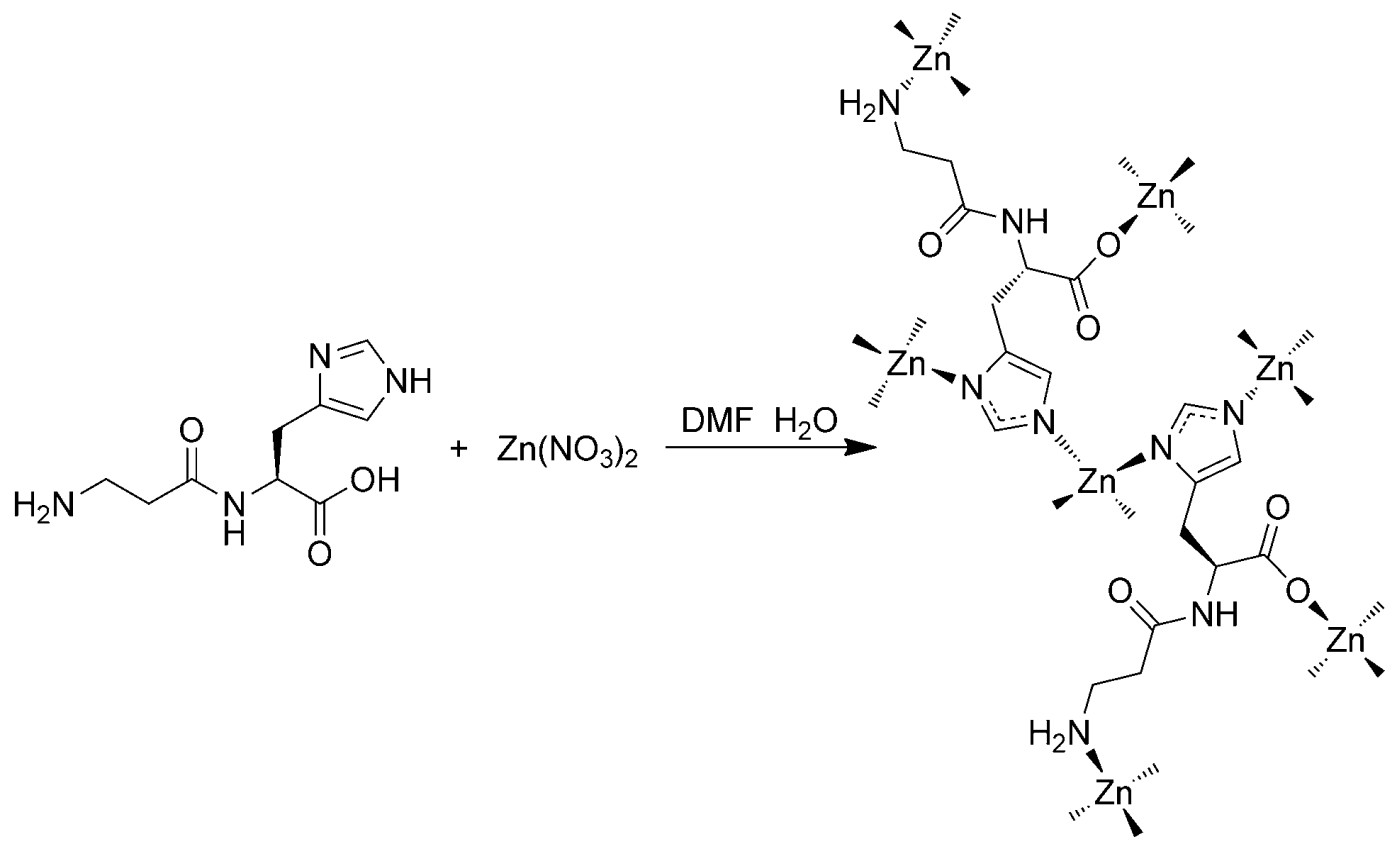
Formation of ZnCar⋅DMF from the reaction between Zn(NO_3_)_2_ and carnosine.

ZnCar⋅DMF crystallizes in the chiral monoclinic space group P2_1_ and its 3D structure is directed by the carnosine conformation (Figure 1 a), as the Zn cations connected to the histidine moiety define a plane and the residue of the N-terminal β-alanine is extended to the third dimension. The imidazolate rings (im) and Zn cations form a zigzag chain where the rings adopt a trans conformation (Figure 1 b) and the Zn-im-Zn angle is 138°(Figure S2), which is smaller than the 145° angle found in ZIFs.[11a] This chain is similar to the linear structures of univalent metal–imidazolate compounds.[13] The chains are linked through the histidine carboxylate group (Figure 1 b) to form undulating layers (parallel to the 100 plane) that are interconnected by the antiparallel-oriented β-alanine residues (Figure 1 c). The framework is further stabilized by two H bonds, an intramolecular bond between the carboxy oxygen atom and amino hydrogen atom of β-alanine, and an intermolecular bond between the carboxylate oxygen atom of histidine and the neighboring amide hydrogen atom (Figure 1 a and c). The arrangement of carnosine forms 1D square-shaped pores, filled with DMF and running parallel to the crystallographic *b* axis (Figure 1 d). The chiral shape of the pore walls is depicted using the Connolly surface representation (Figure 1 e). The pores can be viewed as relatively large cavities, with diameters of *d*_1_=5.18 Å, connected in a zig-zag fashion by narrow channels with diameters of *d*_2_=3.78 Å.

**Figure 1:**
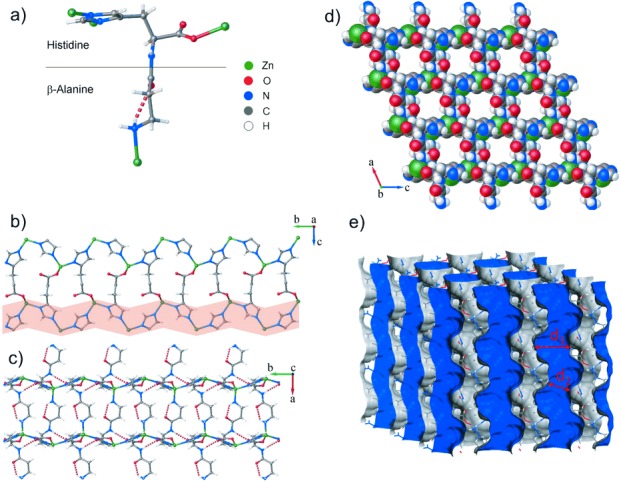
a) 3D conformation of carnosine connected to four Zn cations. The dashed line corresponds to the intramolecular H bond between a carboxy oxygen atom and an amino hydrogen atom. Three Zn cations are bound to histidine (above the line) and the fourth to β-alanine (below the line). b) Zn cations and imidazolate rings form the zig-zag chain (highlighted) that is the rigid part of the structure. The chains are linked through the histidine carboxylate group. c) Undulating layers of imidazolate rings interconnected by antiparallel-oriented β-alanine residues. Intermolecular H bonds are formed between the carboxylate oxygen atom and the amide hydrogen atom. d) Space-filling representation of ZnCar viewed along the b axis, showing the 1D pores. e) Connolly surface representation of the pore walls, calculated with a probe radius of 1.4 Å. The pores consist of cavities with diameters of *d*_1_=5.18 Å and narrow channels with diameters of *d*_2_=3.78 Å.

The structure and the composition of ZnCar⋅DMF, obtained from single-crystal X-ray diffraction data are in good agreement with the characterization results of the bulk sample. The powder XRD pattern perfectly matches the simulated pattern from the single-crystal structure and is indexed to the same unit cell (Figure S3 and Table S1). CHN analysis results are very close to theoretical values (Table S2) and the DMF content, calculated as 20 wt % from the single-crystal data, was verified by thermogravimetric analysis (TGA; Figure S4). The solid-state ^13^C CPMAS NMR spectrum of ZnCar⋅DMF displays all the expected resonances for carnosine and three resonances for DMF (Figure S5) at room temperature.

The ZnCar⋅DMF framework is structurally flexible and displays permanent porosity after the removal of DMF, as demonstrated by the variable-temperature single-crystal X-ray diffraction data. While the crystallinity of the material is retained over the whole temperature range that was studied, the unit-cell volume decreases sharply from 762.82 to 726.78 Å^3^ (by ≈5 %) between 388 and 394 K as DMF is removed from the pores (Figure S6). The relaxation of the ZnCar framework from the DMF-solvated state at 388 K (Figure 2 a) to the desolvated state at 394 K (Figure 2 b) results in an alternate linker conformation. The torsion angles of the peptidic chain change by 20–30° (Figure S7) with the exception of the rigid *ω* angle C7-N8-C9-C14, which corresponds to the peptide bond itself. The structure of the pore was also altered, having cavities with diameters of *d*_1_=4.58 Å connected by channels with diameters of *d*_2_=4.18 Å (Table S3). In contrast, the three torsional angles responsible for the orientation of the imidazolate ring and the carboxylate group on histidine each changed by less than 2°, indicating the rigidity of the *bc* planes shown in Figure 1 b.

**Figure 2:**
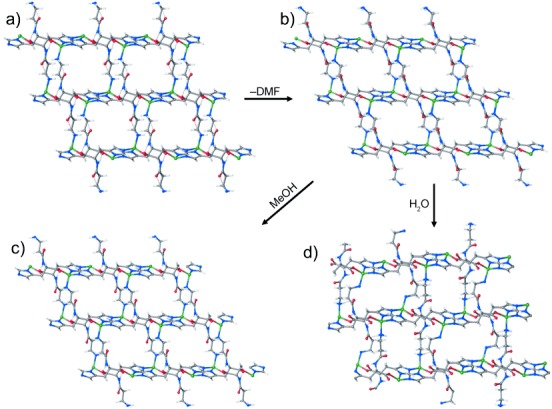
The structures of a) ZnCar⋅DMF, b) desolvated ZnCar, c) resolvated ZnCar⋅MeOH, and d) resolvated ZnCar⋅H_2_O. ZnCar⋅DMF, ZnCar, and ZnCar⋅MeOH show one type of pore with square or parallelogram shape. ZnCar⋅H_2_O has two types of pores.

The bulk desolvation of ZnCar⋅DMF was achieved by evacuation on a high-vacuum line (10^−5^ mbar) at 130 °C for 5 hours. The structure of the evacuated sample, ZnCar, was confirmed by powder XRD and the complete removal of DMF was verified by TGA (Figure S8). The porous properties of desolvated ZnCar were investigated with the CO_2_ adsorption–desorption isotherm (Figure S9) obtained at 195 K up to 1 bar. ZnCar is purely microporous, as the isotherm shape corresponds to that of type I according to the IUPAC classification. The BET surface area is calculated as 448 m^2^ g^−1^ using the relative pressure range, 0.01<*P*/*P*_o_<0.1. The total pore volume, estimated at 0.19 cm^3^ g^−1^, is slightly smaller than the solvent-accessible volume of the desolvated ZnCar structure of 0.21 cm^3^ g^−1^. The isotherm shows no gate opening, but a low pressure hysteresis loop associated with the strong adsorption of CO_2_.

The chemical stability of ZnCar⋅DMF was examined by soaking fresh material in water under stirring for 3 days (Table S4). ICP analysis of the supernatant aqueous solution every 24 hours showed very low Zn leaching, corresponding to 1.7 wt % of the Zn added to water in the form of ZnCar⋅DMF. The stability of ZnCar⋅DMF in water, unlike Zn(Gly-Ala)_2_ and Zn(Gly-Thr)_2_ frameworks, which are stable only in organic solvents, is attributed to the Zn–imidazolate bonding that is the basis of the chemical stability of ZIFs.

To study the stability and the interaction of this framework with different guest molecules, desolvated crystals of ZnCar were immersed in H_2_O and MeOH. The resolvated crystals resulted in two new structures, ZnCar⋅MeOH and ZnCar⋅H_2_O. The ZnCar framework proved to be adaptable to the presence of guest molecules, as the peptide conformation changed in both cases without breaking the 4:4 connectivity motif between Zn and carnosine. ZnCar⋅MeOH adopts the monoclinic *P*2_1_ space group with slightly altered unit-cell parameters, but without significant changes in the pore shape compared to the desolvated structure (Figure 2 c). The torsion angles of carnosine in ZnCar⋅MeOH are in a range between those in the structure containing DMF and the desolvated structure (Figure S7). Using MD simulations, we found that the carboxy oxygen atom of alanine in the desolvated structure is sterically screened from forming an H bond with a methanol molecule (Figure S10). Hence, the main structural change caused by the addition of MeOH is the rotation of the carboxy oxygen atom toward the pore where it can now form an H bond with a guest molecule (each MeOH molecule also works as an acceptor and forms an additional H bond with the amine group) (Figure S11b). In the as-made material, the same carboxy oxygen atom forms an intramolecular H bond with one of the two hydrogen atoms on the amine group, while the DMF molecule is bound to the other hydrogen atom (Figure S11a). The DFT-calculated steepest descent energy minimization of solvent-free ZnCar⋅DMF and ZnCar⋅MeOH unit cells showed that both systems relax to assume the structure of the empty framework. This means that both DMF and MeOH guests distort the framework locally by H bonding without inducing a phase transition in the framework itself (Figure S12). MeOH vapor adsorption on ZnCar, collected at three different temperatures, reaches a maximum of 16 wt % (Figure S13). Desolvation of ZnCar⋅MeOH again produces the ZnCar structure (Figure S14), which is as porous as the original DMF-desolvated ZnCar material (Figure S15), thus demonstrating the repeatability of loading and unloading MeOH from ZnCar.

The structure of the water-resolvated ZnCar⋅H_2_O is different, as carnosine adopts two distinct conformations, carnosine 1 and carnosine 2 (Figure S7), which form different sets of H bonds. Thus, two types of pores are formed, a wide and a narrow one (Figure 2 d), and the volume of the unit cell is doubled compared to those of the other three structures. The positions of the water oxygen atoms in both the narrow and the wide pores were determined from the single-crystal diffraction data with the hydrogen atoms added at calculated positions. The orientations of the water molecules were then refined using DFT calculations (Table S6). The carnosine 2 molecule defines a wide pore space, filled by eight H_2_O molecules per unit cell, and exhibits a conformation similar to those of the MeOH-containing structure, but with the amine group pointing in the opposite direction. Carnosine 2 forms six H bonds (Figure 3 a), three within the framework and three with H_2_O. The narrow pore is filled with four H_2_O molecules and is bound by carnosine 1, in which the torsion angles of the peptidic chain (Figure S7) were significantly changed compared to those of all the other structures (Figure 2 a–c). Carnosine 1 forms three H bonds (Figure 3 b), one within the framework and two with H_2_O. The intraframework H bond between the carboxylate oxygen atom and amide hydrogen atom, present in the three previous structures, appears only in carnosine 2 in ZnCar⋅H_2_O. The H_2_O molecules are interconnected by H bonds forming a network inside the pores. In particular, two out of four distinct H_2_O molecules in the wide pore form the maximum possible four H bonds, two of which are with the framework (Figure S16a). In the narrow pore, there are two distinct H_2_O molecules that form three and two H bonds, respectively (Figure S16b).The desolvated ZnCar structure is obtained after H_2_O removal (Figure S17) and the experimentally measured pore volume, 0.21 cm^3^ g^−1^ (Figure S18), equals to the estimated value from the ZnCar single-crystal structure. H_2_O vapor adsorption isotherms on ZnCar exhibit broad hysteresis loops and a maximum uptake of 15 wt % (Figure S19), and were cycled three times without change. The exhibited reversible resolvation–desolvation of the ZnCar framework with H_2_O proves that the properties of ZnCar remain unaffected after its exposure to water. Unlike ZnCar⋅MeOH and ZnCar⋅DMF, the DFT-calculated energy minimization of the empty ZnCar⋅H_2_O structure did not converge to that of the desolvated material, but relaxed to a different stable state that was 53 kJ mol^−1^ higher in energy (Table S6). This means that the desolvated ZnCar and ZnCar⋅H_2_O structures are separated by an energy barrier, and the calculated energy increase for the framework is counterbalanced experimentally by the formation of the water network that is rich in H bonds. The framework transformations, driven by the different guests, are facilitated mainly by the torsional flexibility of the peptidic chain. The 2D layers constructed from the Zn–imidazolate chains constitute the rigid part of the framework, as indicated by small changes in the Zn-im-Zn angle, the Zn–Zn distance, and the distance between the chains (Table S7).

**Figure 3:**
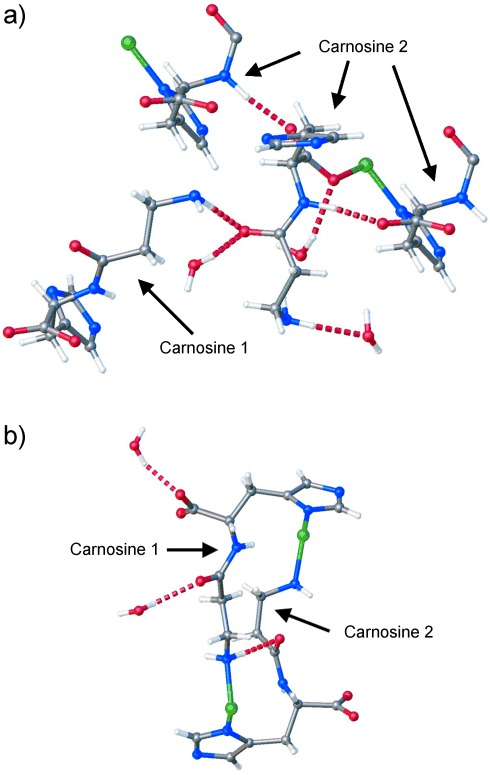
H bonds in ZnCar⋅H_2_O. a) Carnosine 2 has six H bonds in total. Three of them are within the framework, one with carnosine 1 and two with carnosine 2, and the other three with H_2_O. b) Carnosine 1 has three H bonds, one of which is in the framework with carnosine 2 and the other two with H_2_O.

High pressure CO_2_ and CH_4_ adsorption isotherms of ZnCar were collected at 283, 293, and 303 K (Figure S20). The gravimetric uptakes were found to be around 19 wt % for CO_2_ and 5 wt % for CH_4_ at 15 bars. The isotherms are of type I, and in each case 80 % of total uptake was adsorbed at 4 bars, showing the entirely microporous character of this material. The ZnCar MOF exhibits strong binding affinity for both gases, as shown by the isosteric heat of adsorption *Q*_st_ (Figure S21). For CO_2_, the *Q*_st_ value at zero coverage is 49 kJ mol^−1^, which is amongst the highest ever reported for MOFs. Cu-BTTri-mmen exhibits a *Q*_st_ value of 96 kJ mol^−1^,[14] and for the most microporous MOF, the *Q*_st_ at zero coverage ranges from 25–50 kJ mol^−1^.[15] Our DFT calculations indicate that most of this energy can be attributed to the dispersion interactions (Table S8), in particular between the imidazole ring and the CO_2_ carbon atom (Figure S22). The molecular dynamics (MD) simulations of CO_2_ diffusion along the pores have also shown this configuration to be the most favorable for adsorption, and showed that the CO_2_ molecules diffuse by hopping between the cavities in the pore structure through smaller channels that connect the pores (Figure 1 e and Figure S22). The *Q*_st_ for CH_4_ adsorption at zero coverage is 27 kJ mol^−1^, which is also a high value for MOFs,[16] very close to the highest reported *Q*_st_ value 30 kJ mol^−1^ for PCN-14.[17]

The selectivity of adsorption between CO_2_ and CH_4_ was estimated using the IAST model at 303 K for the equimolar gas mixture, and shows a value of 10 for the whole pressure range (Figure S23). This is a moderate value for MOFs, lower than that of Mg_2_(dobdc), which is 60,[18] in accordance with the high adsorption affinity of ZnCar for both gases.

The β-dipeptide carnosine is a special MOF linker that offers chemical stability, structural diversity and chirality, and permanent porosity in the ZnCar⋅DMF compound. ZnCar⋅DMF is an analogue of ZIFs where the involvement of the imidazole ring of the histidine side chain in Zn–imidazolate bonding both affords the chemical stability of the framework in water and produces a 3D structure, in contrast to previously studied peptide-based MOFs. The main His-β-Ala chain connects the rigid imidazolate-based layers, retaining its flexibility and conferring structural adaptability on the framework in the presence of different guest molecules. The ZnCar framework exhibits permanent microporosity and strong adsorption affinity for CO_2_ and CH_4_.
