# Supplementary material for: Guest-Adaptable and Water-Stable Peptide-Based Porous Materials by Imidazolate Side Chain Control
Source: Angew Chem Int Ed Engl. 2013 Dec 2;53(1):193–8. doi: 10.1002/anie.201307074 (PMC3995008; doi:10.1002/anie.201307074)
Supplement: Supplementary file 1 [file anie0053-0193-sd1.pdf]

Angewandte Chemie
Eine Zeitschrift der Gesellschaft Deutscher Chemiker

Supporting Information
© Wiley-VCH 2013
69451 Weinheim, Germany

Guest-Adaptable and Water-Stable Peptide-Based Porous Materials by Imidazolate Side Chain Control**

*Alexandros P. Katsoulidis, Kyo Sung Park, Dmytro Antypov, Carlos Martí-Gastaldo, Gary J. Miller, John E. Warren, Craig M. Robertson, Frédéric Blanc, George R. Darling, Neil G. Berry, John A. Purton, Dave J. Adams, and Matthew J. Rosseinsky**

anie_201307074_sm_miscellaneous_information.pdf

Supporting information

Table of Contents

SI 1 Experimental section……………………………………………………….…………….……4

SI 2 Characterization……………………………………………………………..…………5

SI 3 Theoretical section…………………………………………………………..…………..10

Table S1 Selected crystallographic details and refinement parameters for the four Zn carnosine systems…………………………………………………………………………………..………12

Figure S1 SEM images of ZnCar•DMF crystals……………………………........................13

Figure S2 The Zn-im-Zn angle in ZnCar•DMF……………………………………………14

Figure S3 Powder XRD pattern of as made ZnCar•DMF………………………...…………15

Table S2 CHN analysis of ZnCar•DMF……………………………………..………...…16

Figure S4 Thermogravimetric analysis of ZnCar•DMF under air flow…………………..…..17

Figure S5 Solid state ^{13}C CPMAS NMR of ZnCar•DMF…………………...........................18

Figure S6 Unit cell dimensions and volume of ZnCar•DMF from VT single crystal experiments……………………………………………………………………………......19

Figure S7 Torsion angles of ZnCar•DMF, ZnCar, ZnCar•MeOH and ZnCar•H_2O…………………………………………………..………........................20

Table S3 Geometric characteristics of the 1D pores in ZnCar frameworks……………......…21

Figure S8 Powder XRD and thermogravimetric analysis of desolvated ZnCar......................22

Figure S9 CO_2 adsorption desorption isotherm of ZnCar at 195 K……………….………....23

Table S4 ICP analysis of Zn after water stability test of ZnCar•DMF…………………..….24

Figure S10 Snapshots from MD simulations of ZnCar framework with MeOH……………………………………………………………………….……………......25

Figure S11 H-bonds in ZnCar•DMF and ZnCar•MeOH………….…….………………….26

Figure S12 The evolution of the unit cell volume of ZnCar•DMF, ZnCar and ZnCar•MeOH during energy minimization with DFT…………………….…………..…….………..……27

Table S5 Unit cell parameters of DFT minimized structures for ZnCar•DMF, ZnCar and ZnCar•MeOH……………………………………..…………………….……..27

Figure S13 MeOH vapor adsorption desorption isotherms of ZnCar at 10, 20 and 30°C……28

Figure S14 Powder XRD and thermogravimetric analysis of desolvated ZnCarH2O..............29

Figure S15 CO_2 adsorption desorption isotherm of desolvated ZnCar•MeOH at 195 K……..30

Figure S16 H_2O network in the wide and the narrow pore of ZnCarH2O…………………....31

Figure S17 Powder XRD and thermogravimetric analysis of desolvated ZnCarH2O..............32

Figure S18 CO_2 adsorption desorption isotherm of desolvated ZnCar•H2O at 195 K……….33

Figure S19 H_2O vapor adsorption desorption isotherms of ZnCar at 20, 25 and 30°C………34

Table S6. DFT results for ZnCar•H_2O…………………………………….…..…………….35

Table S7 Characteristic angles and distances within the framework……………..………..…36

Figure S20 High pressure CO_2 and CH_4 adsorption isotherms of ZnCar…...…..……………37

Figure S21 Isosteric heat of CO_2 and CH_4 adsorption on ZnCar……...……...…….…………38

Table S8 Cell parameters without and with VDW correction…………………….……...……39

Table S9 The energy values in eV for guest molecules in the gas phase……….………..……39

Figure S22. Equipotential surfaces calculated of ZnCar without and with CO_2 by DFT……………………………………………………………………….….…..……...40

Figure S23 Adsorption selectivity of CO_2 over CH_4 on ZnCar at 303 K estimated with IAST………………………………………………………………………………..41

Figure S24 Positions of guest molecules calculated in 15 ns MD runs at 298 K …..…………42

Table S10. Estimated residence time for diffusion in bulk and across the interface…………..43

Table S11. Estimated time required to empty a 1mm-long sample……………………………44

References……………………………………………………………………...……….45

SI 1 Experimental section.

Materials and reagents. $Zn(NO_3)_2•6H_2O$ (99%) was purchased from Sigma – Aldrich, Carnosine from TCI UK (98%), methanol and DMF from Fisher Chemical. All reagents and solvents were used without any previous purification.

Synthesis of ZnCar•DMF. 5.26 ml of $Zn(NO_3)_2•6H_2O$ aqueous solution (0.336 M), 2ml of carnosine aqueous solution (0.442 M), 2 ml of water and 20 ml of DMF were loaded into a 40 ml scintillation vial. The vial was heated at 100°C for 12 h with ramping rate 1°C/min and was cooled down to room temperature at 1°C/min. After this time needle-shaped crystals covered the wall and the bottom of vial. The product was collected with filtration and washed thoroughly with methanol. Yield ~ 70%.

Desolvated ZnCar. DMF was removed from the pores of ZnCar•DMF on a high vacuum line. 200 mg of freshly prepared sample were transferred to a tube with a Youngs tap and connected to vacuum line. Vacuum was applied with a mechanical pump (10^{-3} mbar) and the sample was heated to 130 °C. After 2 h the applied vacuum was switched to a turbomolecular pump line (10^{-5} mbar) and held for 5 h.

Resolvated ZnCar•MeOH and ZnCar•H_2O. Desolvated ZnCar crystals were placed in 10 ml two scintillation vials. The first vial was filled with MeOH and the second one with H_2O. After three days the crystals were collected with filtration.

Water stability tests. In three separate vials were mixed 35 mg of freshly prepared ZnCar•DMF and 15 ml of H_2O. The mixtures let under stirring for 1, 2 and 3 days respectively. The solids were separated from the supernant solution with centrifuge. The Zn content of the supernant solutions was analyzed with ICP and expressed as % wt of the total added Zn in the form of ZnCar•DMF.

SI 2 Characterization.

Elemental analysis. Carbon, nitrogen and hydrogen contents were determined by microanalytical procedures using a Thermo EA1112 Flash CHNS-O Analyzer.

Thermogravimetric analysis was carried out with a SEIKO S – II instrument in the 25-650 ºC temperature range under a 5 ºC.min^{-1} scan rate and an air flow of 50 mL.min^{-1}.

X-ray single-crystal data collection and analysis.

Single-crystal X-ray diffraction data for ZnCar•DMF, ZnCar•MeOH and ZnCar•H_2O were collected for suitable samples mounted on a Rigaku AFC-12K goniometer using a Rigaku Saturn 724+ area detector and 007HF Molybdenum rotating anode source. Samples were mounted under inert oil on Mitegen tips and held at 100K under the nitrogen flow from an Oxford Cryosystems Cryostream Plus.[1] Data were integrated using SAINT[2] and scaled using SADABS[3]. Structures were solved by direct methods using SHELXS[4] implemented in Olex2[5] and refined on F_o^2 by full-matrix least squares refinement using SHELXLMP. Non-hydrogen atoms were refined anisotropically. For ZnCar·DMF the hydrogen atoms were located from the Fourier difference map and their parameters were fully refined. For ZnCar·MeOH and ZnCar·H_2O all hydrogen atoms were placed in idealized positions [C—H = 0.99 (CH_2), 0.95(sp^2 CH), 1.00Å (sp^3 CH), 0.84 Å (OH), 0.88 Å (NH) and 0.92 Å (NH_2)] and refined in riding modes with U_{iso}(H) = 1.2U_{eq}(C), 1.2U_{eq}(N) and 1.5U_{eq}(O). In ZnCar·MeOH the disordered methanol molecule was modeled with the carbon atom in two positions with the occupancies set to 0.45 and 0.55. The C-O bond lengths were restrained to be equal to within 0.02 Å using the SADI command and the anisotropic displacement parameters of the two carbon atoms were restrained to be the same using EADP with an ISOR command applied to the carbon and oxygen atom positions. The hydrogen atoms were modeled using HFIX 33 for the methyl hydrogens and HFIX 83 for the hydroxyl hydrogen, which was modeled in two positions to account for the different H-O-C geometries resulting from the two carbon atom positions. In ZnCar·H_2O the hydrogen atom positions for the water molecules could not be located from the Fourier difference map and were added in a simplified tetrahedral geometry using AFIX 6 as implemented by the hydrogen atom placement procedure in Olex2. These hydrogen atoms were included in the model for subsequent use in the DFT energy minimization calculations described below and cannot be considered to be an accurate structural model.

Variable temperature single crystal diffraction experiment details

Course Variable Temperature Experimental

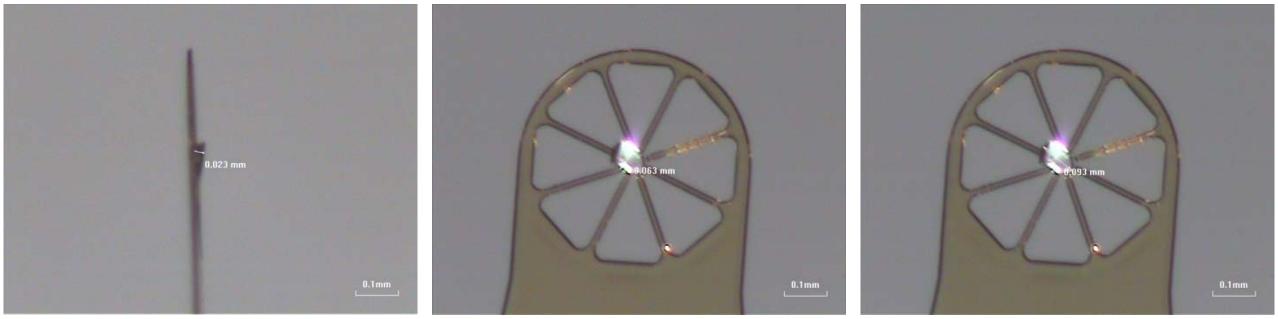

Single crystals of ZnCar•DMF were isolated with motherliquor from the reaction vessel and placed on a microscope slide. A regular colourless prismatic crystal was isolated, 23x63x92 μm in size from an array of differently sized and shaped colourless crystals. The crystal was dry mounted to a 50 μm Mitegen Gripper with no oil and placed under the nitrogen flow of an Oxford Cryosystem 700+[1] at 100 K on a Rigaku AFC12K goniometer.

Data were collected using a Saturn 724+ detector and 007HF Molybdenum microfocus rotating anode X-ray source. The crystal was kept at 100 K for the duration of the ground state experiment. Once the crystal quality was confirmed from the ground state data a variable temperature experiment was setup using CCVT[6]. The crystal was heated at at ramp rate of 350 K/hr with a 5 K variance and 10 minute dwell for all experiments. A full data collection was undertaken at each temperature step comprising of 5 ω-scans at 0.5º slicing were undertaken. Three 125° ω-scans at 0, 120, 240° settings in φ and common setting of -68° in κ followed by a single 125° scan at κ = -30° and φ = 90° at a counting time of 10 seconds per frame with the final low angle 180° ω-scan at κ = φ = 0° where a counting time of 4 seconds per frame was employed.

The sample was initially heated in 100 K steps from 100 to 300 K. Then from 300 to 400 K in 20 K increments. The incremental step was reduced for the final ramp from 400 to 450 K to 10 K per step. The sample was then cooled back to 100 K where two data collections were undertaken before the sample was heated to 500 K and subsequently cooled back to 100 K for the final data collection.

Fine Variable Temperature Experimental

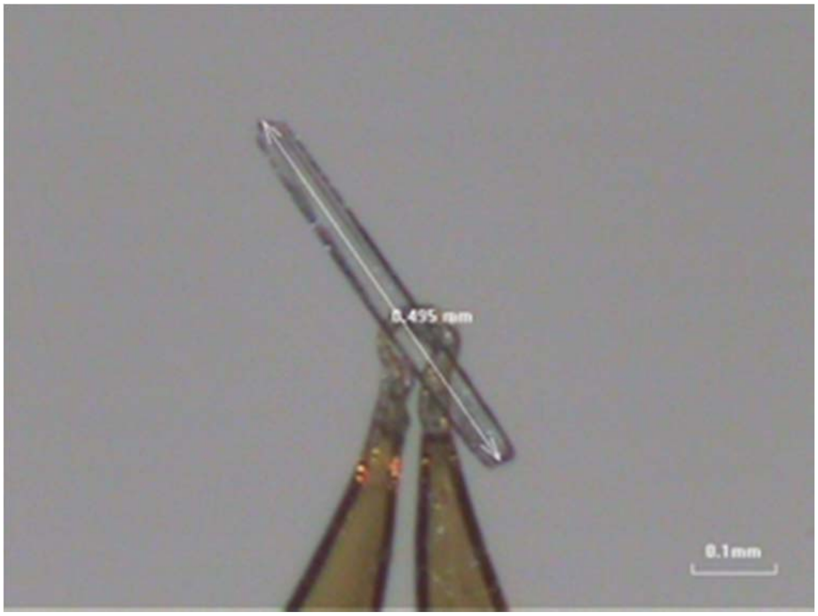

A large colourless rectangular prismatic crystal was isolated as per the procedure defined previously. The crystal was glue mounted to 50 µm Mitegen tip due its shape being incompatible with the gripper used for the VT experiment.

As previously described the crystal was kept at 100 K for the duration of the ground state experiment. Once the crystal quality was confirmed from the ground state data a variable temperature experiment was setup using CCVT. The crystal was heated at at ramp rate of 330 K/hr with a 1 K variance and 10 minute dwell for all experiments. A full data collection was undertaken at each temperature step comprising of 5 ω-scans at 0.5º slicing were undertaken. Three 125° ω-scans at 0, 120, 240° settings in φ and common setting of -68° in κ followed by a single 125° scan at κ = -30° and φ = 90° at a counting time of 5 seconds per frame with the final low angle 180° ω-scan at κ = φ = 0° where a counting time of 2 seconds per frame was employed.

The sample was initially heated in a 140 K step from 100 to 240 K. Then from 240 to 340 K in an 100 K increment. Then 340 to 380 K in 20 K steps. The incremental step was reduced for the final ramp from 380 to 408 K to 2 K per step. Unfortunately due to a technical fault in the Cryostream the variance at each step could exceed the allowed 1 K as observed in the figure below. When this happened the data collection automatically paused resulting in longer data collection times at each temperature step. The crystal alignment was checked at 408 K to confirm the sample mount stability.

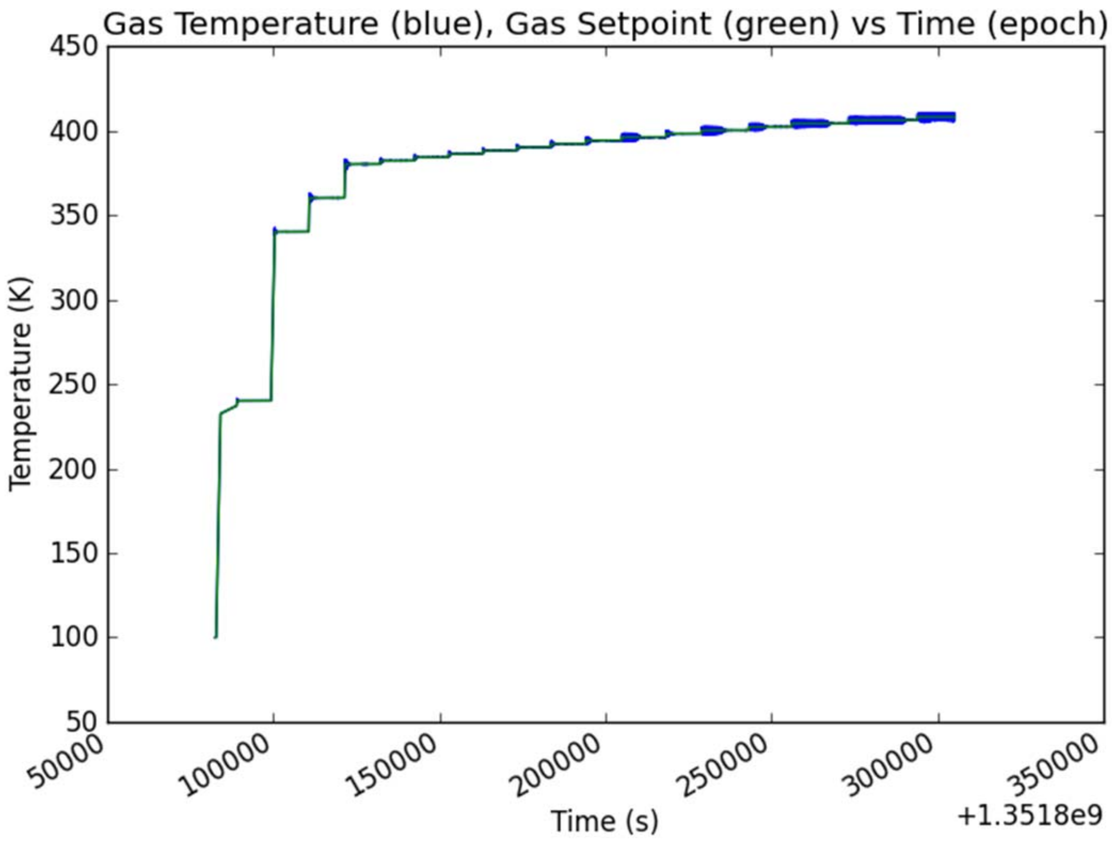

Initial Variable Temperature Processing

Unit cell and temperature parameters were automatically extracted from the data collection using the programSTANAL.[7] Experimental data collection parameters were checked using CCVT2LOG.[8]

All the collected Rigaku images were converted using FMD[9] into Bruker format images and subsequently processed using APEXII[10] and integrated using SAINT[2] and scaled using SADABS[3]. All the data was initially examined using Olex2[5] the structures being solved with the olex2.solve structure solution program[11] using Charge Flipping and refined with the ShelXL_ifc refinement package[4] using Least Squares minimisation.

X-ray powder diffraction XRPD patterns were collected in transmission geometry at 298 K by using a STOE Stadi-P diffractometer with Cu Kα radiation (λα = 1.54184 Å). Samples were ground and transferred to a 0.5 mm internal diameter glass capillary. Typically, profiles were collected as step scans over a 1.5 h period in the 5º< 2Θ< 30º range with a step size of 0.25º.

^{13}C CPMAS Solid state NMR. Solid-state NMR experiments were performed on a 9.4 T Bruker DSX NMR spectrometer equipped with a 4 mm HXY triple-resonance MAS probe (in double resonance mode) tuned to ^{13}C at $\nu_0(^{13}C)$ = 100.53 MHz. Experiments were performed at room temperature under magic angle spinning (MAS) at frequency rate of□ν_r = 10 kHz. ^{1}H pulses and SPINAL-64 heteronuclear decoupling[12] were performed at a radio-frequency (rf) field amplitude of $\nu_1(^1H)$ = 83 kHz. ^{13}C spectra were recorded under ^{1}H ^{13}C cross polarisation (CP) condition with a ^{13}C rf field of $\nu_1(^{13}C)$ = 50 kHz, while the ^{1}H rf field amplitude was ramped to obtain maximum signal at approximately $\nu_1(^1H)$ = 60 kHz. Recycle delay of 10 s and contact time for CP of 500 μs were used. ^{13}C chemical shifts were referenced to tetramethylsilane (δ_{iso} = 0 ppm) by using CH_2 group of adamantane at 29.45 ppm as a secondary reference.[13]

SEM. Crystals of ZnCarDMF were photographed in a Hitachi S-4800 Field-Emission Scanning Electron Microscope. The crystals were previously sputter coated with gold.

Gas and Vapor Sorption Measurements. The gas and vapor isotherms of ZnCar with N_2, CO_2,CH_4, MeOH and H_2O were measured by using an Intelligent Gravimetric Analyzer (IGA) from Hiden. The samples were activated at 130º C overnight before each data collection. Isotherms for CO_2 were measured at 195, 283, 293 and 303 K, at 283, 293 and 303 K for CH_4, at 77 K for N_2, at 283, 293 and 303 K for MeOH and at 293, 298 and 303 K for H_2O. A measurement "time-out" of 40 minutes was used for all measurements, this being the maximum time allowed for the measurement of a single data point. The specific surface area was calculated applying the BET equation on the CO_2 adsorption data at 195 K in the range $0.01<P/P_o<0.1$. The range of pressure was selected according the consistency criteria of Rouquerol.[14] The total pore volume was calculated from the adsorbed CO_2 at 1 bar – 195 K. The isosteric heat of adsorption for CO_2 and CH_4 was estimated by applying the virial equation method to the adsorption data at three temperatures (283 K, 293 K and 303 K).[15] The IAST method was applied for the adsorption selectivity using the CO_2 and CH_4 adsorption data at 303 K.[16]

SI 3 Theoretical section.

Density Functional Theory (DFT). All 3D-periodic DFT calculations of a single unit cell containing 4 linkers and 2 zinc cations were performed with the Vienna ab initio Simulation Package (VASP)[17] version 5.2.12. The PBE functionals were used in the framework of Projector Augmented Wave method[18] and the Generalised Gradient Approximation.[19] The specific settings used for the VASP calculations were:

- Gaussian smearing was used with smearing width of 0.1 eV.
- The "accurate" precision setting was used with convergence criteria of $2x10^{-5}$ eV for the electronic energy convergence and 10^{-3} eV/Å for the residual force for the ionic convergence.
- Standard and hard potentials were tested with respectively 600 eV and 900 eV energy cut-offs respectively to produce very similar cell structures. The 600 eV was used for all results presented in this paper
- The method of conjugate gradients was used to optimise the ion positions, cell volume and cell shape, with force scaling factor of 0.1. The simulations were typically run for the total of 1000 ionic relaxation steps and periodically restarted to remove the accumulative error in energy arising from varying the cell dimensions.
- The Brillouin zone was sampled using a 2×2×2 Monkhorst-Pack sampling grid
- To take into account van der Waals interactions we used vdW-DF method[20] with non-local exchange-correlation functional revPBE.

All DFT calculations were performed using the UK's national high-performance computing service HECToR provided by UoE HPCx Ltd at the University of Edinburgh, Cray Inc and NAG Ltd and funded by the Office of Science and Technology through EPSRC's High End Computing Programme. The access to HECToR facilities was via our membership of the UK's HPC Materials Chemistry Consortium funded by EPSRC (EP/L000202).

Molecular Dynamics (MD). MD simulations of a typically 4x4x4 super cell containing 128 linkers and 128 zinc cations and guest molecules were conducted in the constant NVT ensemble using NAMD package.[21] The heavy atoms in the framework were fixed at their experimental positions while the hydrogens and the guest molecules were mobile. The temperature was kept constant by Langevin thermostat with damping coefficient 0.1, the simulation time step was 1fs and a typical MD run was 10 ns.

We used the CHARMM22 force-field designed for protein modelling in combination with the Cationic Dummy Atom (CaDA) model[22] to represent zinc cations. In CaDA model, the zinc ion is represented by 4 dummy charges connected by springs and surrounded by a repulsive shell to

account for the excluded volume interactions. Such representation, unlike a simple 1-site model, produces a more realistic description of the electrostatic filed around tetrahedrally-coordinated zinc and also allows for polarization effects to take place.

The initial configurations for the MD simulations were prepared from experimental structures by constraining all heavy atoms to their experimental positions and letting the hydrogens and guest molecules reach thermal equilibrium at a given temperature. The hydrogens on the framework were kept mobile for three reasons. First, because of often large uncertainties in their positions in the XRD. Second, due to a known mismatch between the maximum electron density for light atoms and their geometrical center (e.g. the C-H bond appears shorter in the XRD than its equilibrium length in MD). Third, to provide a better thermal coupling with the guest molecules.

For solvated structures, the known number of solvent (or guest) molecules were placed in each individual pore at random positions, energy minimized to remove high-energy overlaps and then equilibrated at a high temperature while keeping the framework rigid. This was followed by annealing the temperature to 298K and allowing the system to equilibrate for at least 100ps. The equilibrium properties such as local density and the diffusion coefficients were than collected over a time of 10ns.

Table S1 Selected crystallographic details and refinement parameters for the four Zn carnosine systems.

Identification code	ZnCar•DMF	ZnCar	ZnCar•MeOH	ZnCar•H_2O
Empirical formula	$C_{12}H_{19}N_5O_4Zn$	$C_9H_{12}N_4O_3Zn$	$C_{10}H_{16}N_4O_4Zn$	$C_{18}H_{36}N_8O_{12}Zn_2$
CCDC Number	949241	949242	949243	949244
Formula weight	362.69	289.60	321.64	687.29
Temperature/K	100	394(2)	100	100
Crystal system	Monoclinic	Monoclinic	Monoclinic	Monoclinic
Space group	$P2_1$	$P2_1$	$P2_1$	$P2_1$
a/Å	9.3427(5)	9.198(3)	9.2522(8)	10.906(2)
b/Å	9.1269(5)	9.330(3)	9.2556(7)	8.9687(17)
c/Å	9.6040(5)	9.636(3)	9.4781(8)	14.585(3)
α/°	90.00	90.00	90.00	90.00
β/°	114.455(2)	118.480(11)	118.419(3)	96.631(12)
γ/°	90.00	90.00	90.00	90.00
Volume/$Å^3$	745.46(7)	726.8(4)	713.84	1417.3(5)
Z	2	2	2	2
$\rho_{calc}mg/mm^3$	1.616	1.323	1.496	1.611
m/mm^{-1}	1.674	1.691	1.735	1.763
F(000)	376.0	296.0	332.0	712.0
Crystal size/mm^3	0.100 x 0.055 x 0.050	0.495x 0.049 x 0.012	0.1 × 0.02 × 0.015	0.07 × 0.02 × 0.01
2Θ range for data collection	4.66 to 52.9°	4.8 to 41.78°	4.88 to 54°	3.76 to 52.84°
Index ranges	-11 ≤ h ≤ 11, -11 ≤ k ≤ 8, -12 ≤ l ≤ 11	-9 ≤ h ≤ 9, -9 ≤ k ≤ 9, -9 ≤ l ≤ 9	-11 ≤ h ≤ 11, -11 ≤ k ≤ 8, -12 ≤ l ≤ 12	-11 ≤ h ≤ 13, -11 ≤ k ≤ 8, -18 ≤ l ≤ 17
Reflections collected	11480	7194	10516	15193
Independent reflections	2798 [R(int) = 0.0484]	1543 [R(int) = 0.0833]	2820 [R(int) = 0.0992]	5464 [R(int) = 0.1162]
Data/restraints/ parameters	2798/1/209	1543/1/154	2820/38/176	5464/1/380
Goodness-of-fit on F^2	1.030	1.066	0.985	0.953
Final R indexes [I>=2σ (I)]	R_1 = 0.0264, wR_2 = 0.0608	R_1 = 0.0561, wR_2 = 0.1324	R_1 = 0.0487, wR_2 = 0.0994	R_1 = 0.0575, wR_2 = 0.0974
Final R indexes [all data]	R1 = 0.0278, wR2 = 0.0613	R_1 = 0.0714, wR_2 = 0.1423	R_1 = 0.0755, wR_2 = 0.1072	R_1 = 0.1216, wR_2 = 0.1188
Largest diff. peak/hole / e $Å^{-3}$	0.45/-0.71	1.00/-0.38	0.60/-0.56	0.73/-0.68
Flack parameter	0.030(11)	0.02(4)	0.00(3)	0.04(2)

CCDC nnnnnn contains the supplementary crystallographic data for this paper. These data can be obtained free of charge from The Cambridge Crystallographic Data Centre via www.ccdc.cam.ac.uk/data_request/cif using the CCDC deposition numbers shown in the table.

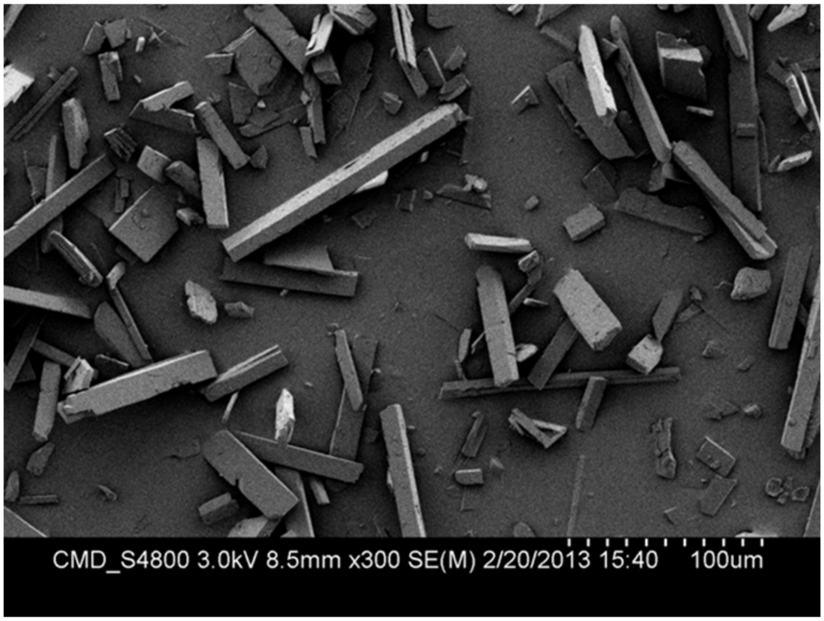

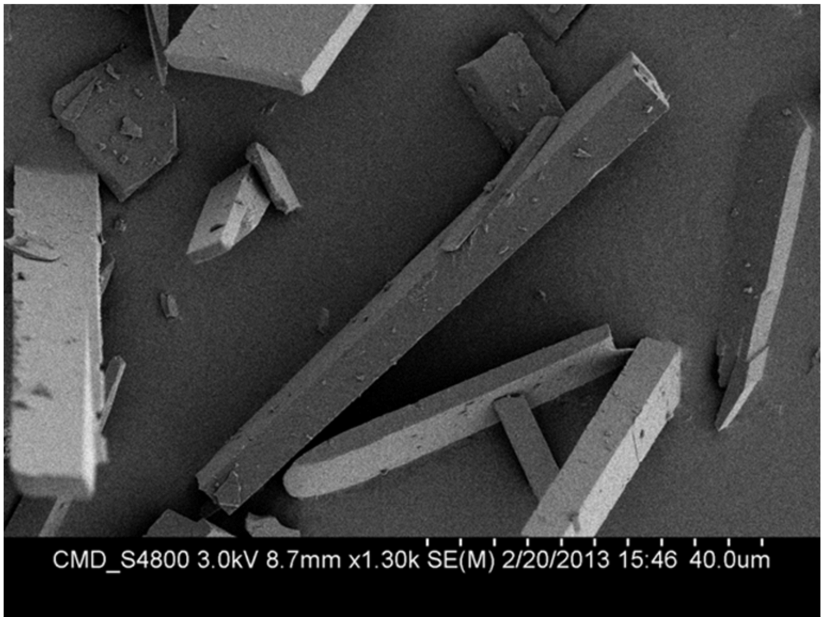

Figure S1 SEM images of ZnCar•DMF crystals show their rectangular prism shape

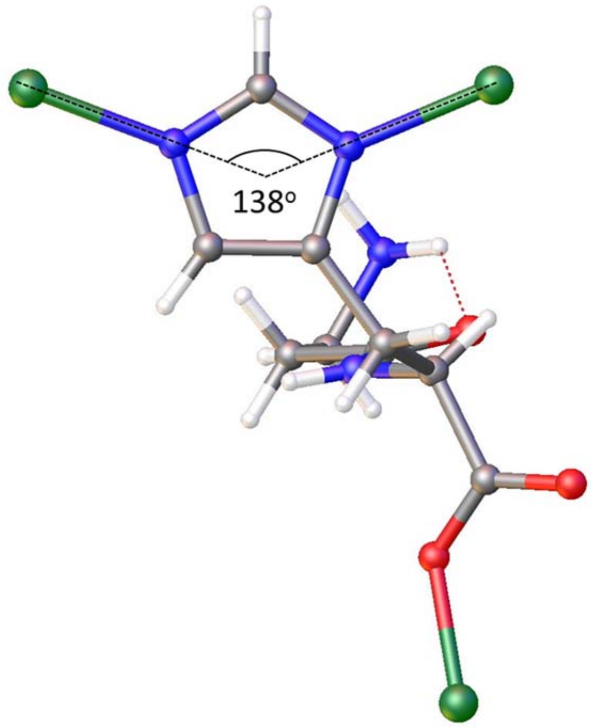

Figure S2 The Zn-im-Zn angle in ZnCar•DMF

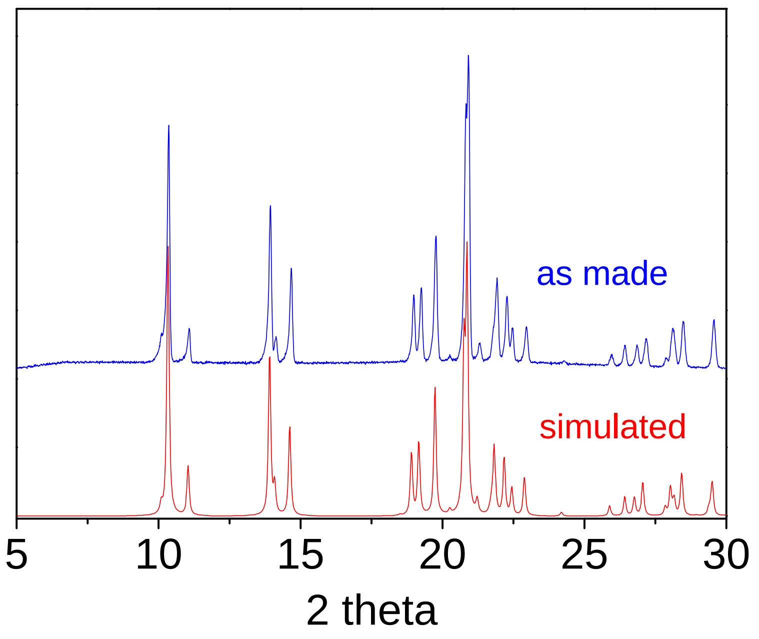

Figure S3 Powder XRD pattern of as made ZnCar•DMF (blue) matches perfectly with the simulated pattern of the single crystal structure of ZnCar•DMF (red)

Table S2 CHN analysis of fresh ZnCar•DMF sample compared to the theoretical composition

	Theoretical ZnCar•DMF	Sample ZnCar•DMF
C	39.73	39.57
H	5.28	5.25
N	19.31	19.17

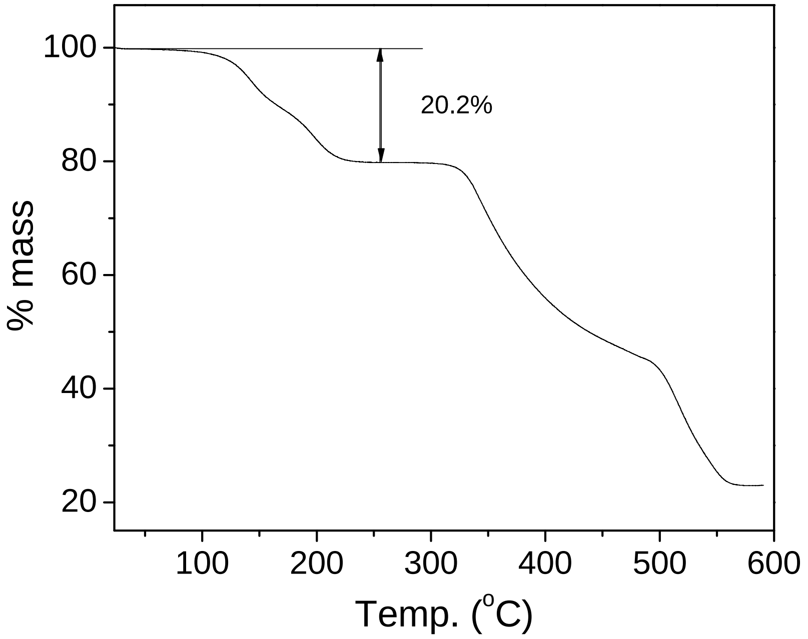

Figure S4 Thermogravimetric analysis of ZnCar•DMF under air flow. The mass loss between 100 and 250°C corresponds to removal of DMF and equals to theoretical content of DMF (20.15%).

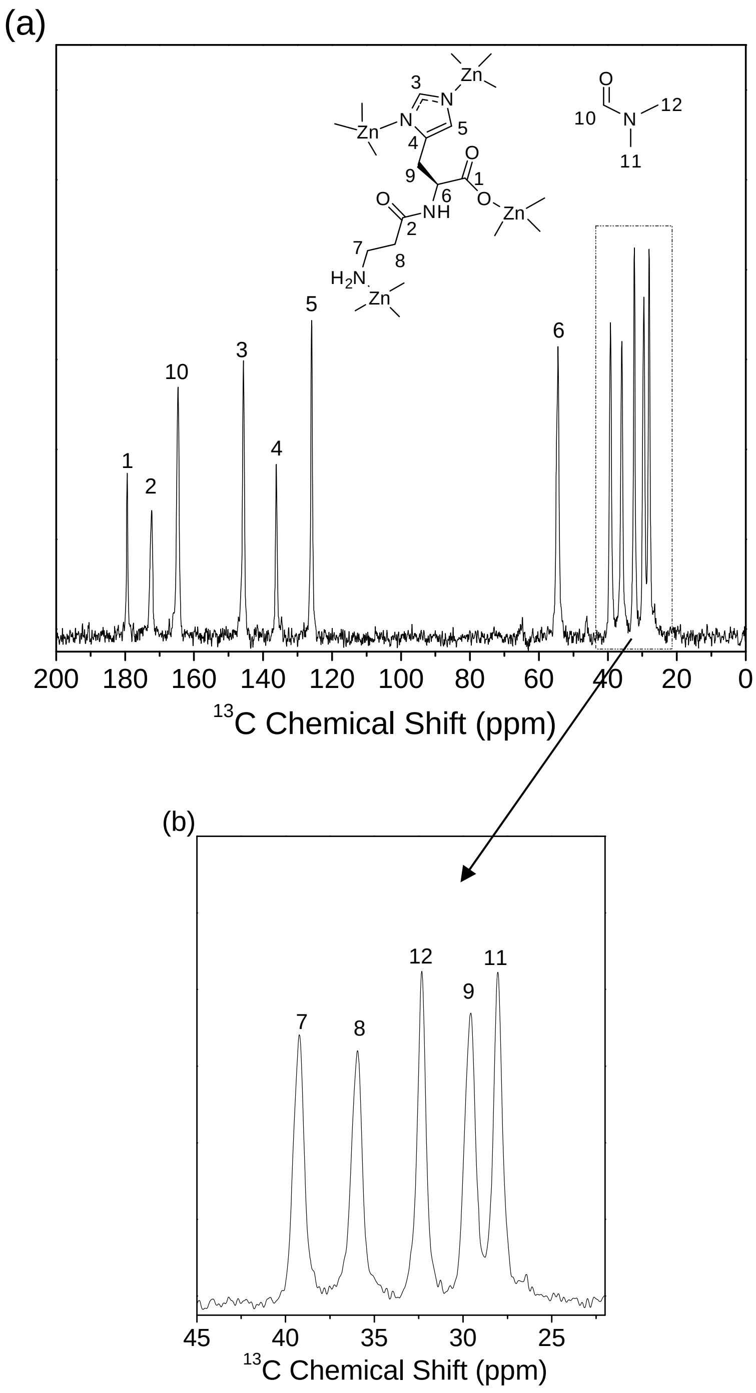

Figure S5 (a) Solid state ^{13}C CPMAS NMR of ZnCar•DMF. (b) The part of the spectrum between 22 and 45 ppm is shown in the second graph.

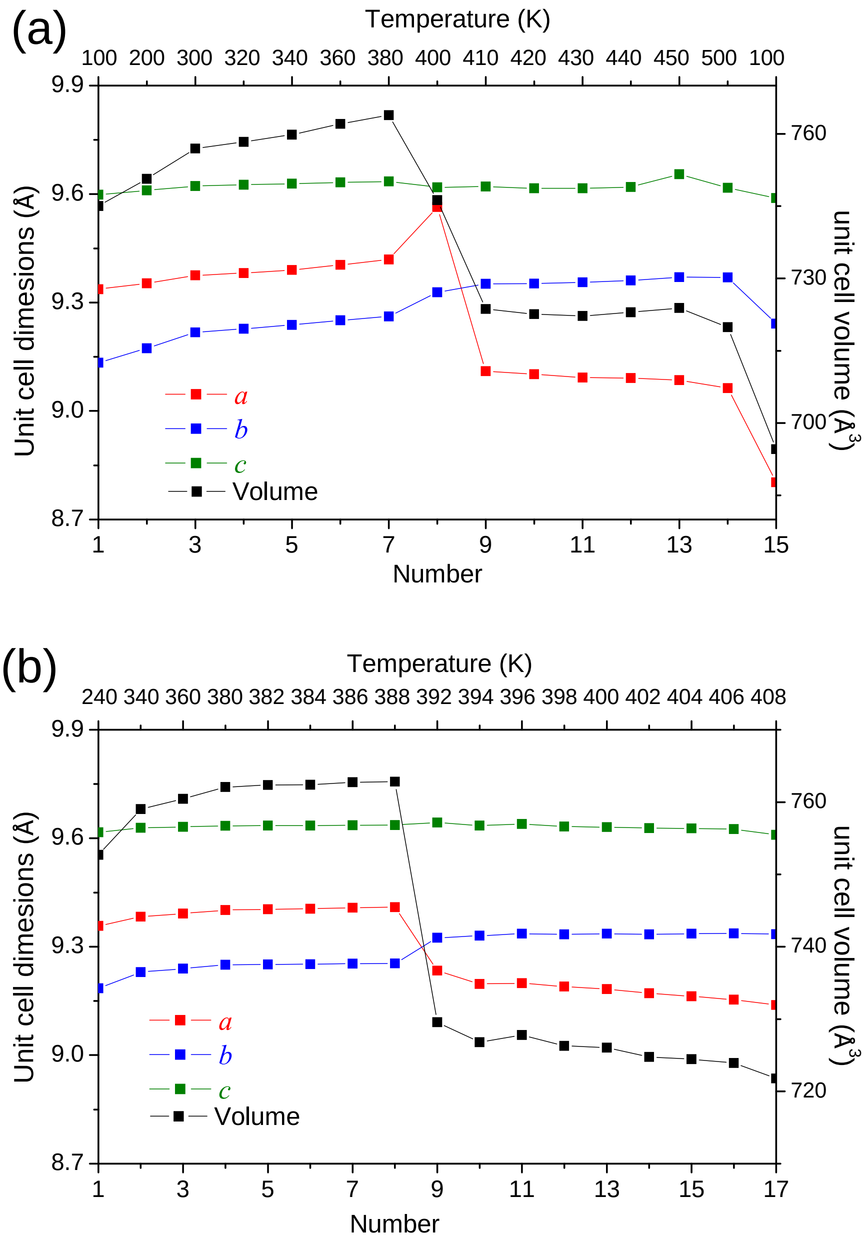

Figure S6 (a) Unit cell dimensions and volume of ZnCar•DMF from automated variable temperature diffraction data collection from 100 to 500 K. (b) Unit cell dimensions and volume of ZnCar•DMF in a narrower temperature range, from 380 to 408 K the data collected every 2 K.

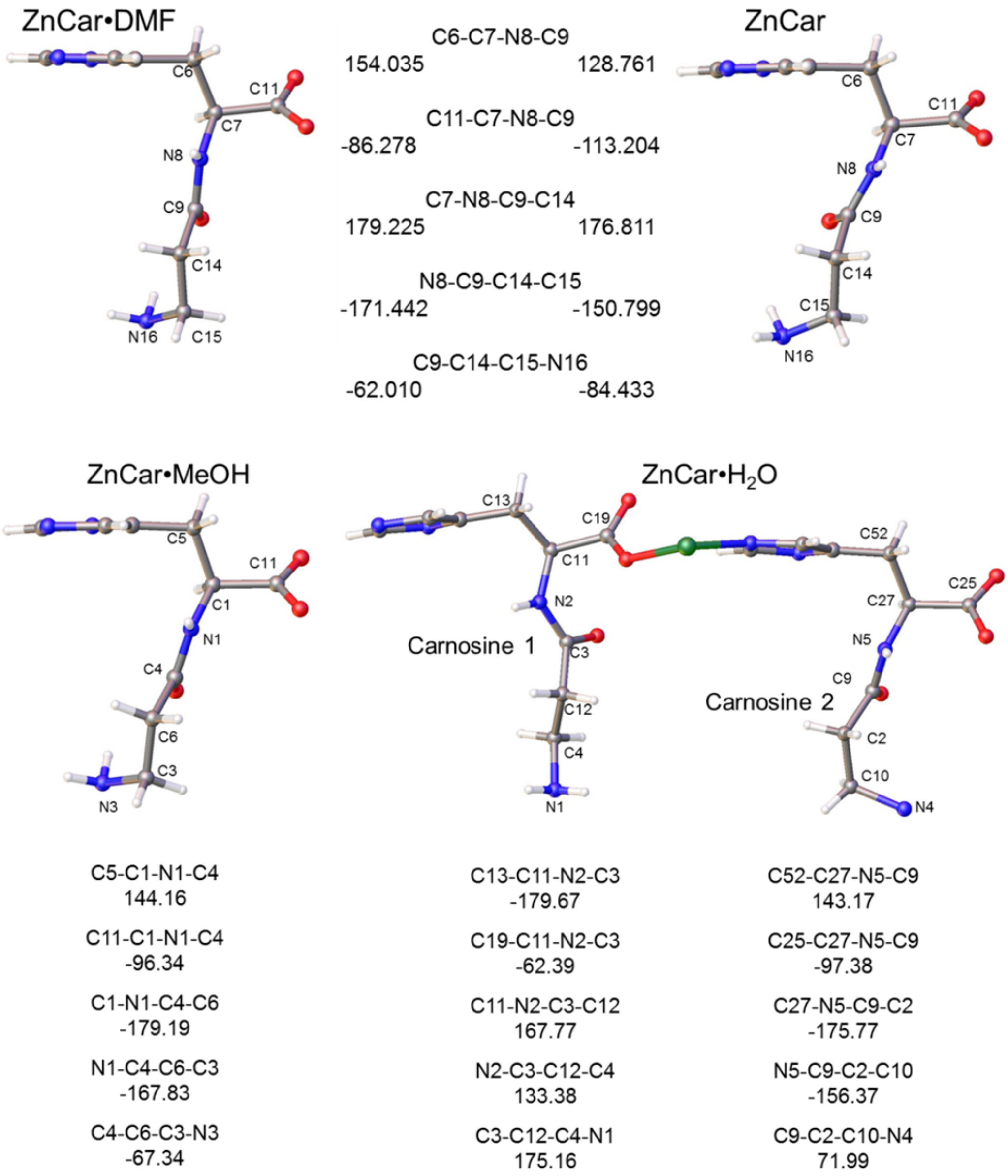

Figure S7 Torsion angles of most flexible part of ZnCar•DMF at 388 K (top left), ZnCar at 394 K (top right) and ZnCar•MeOH (bottom left). Torsion angles of two distinct carnosine molecules in ZnCar•H_2O (bottom right).

Table S3. Geometric characteristics of the 1D channels in solvated and desolvated Zn-carnosine frameworks calculated with olex2 for structures refined from the SCXRD data. Here d_1 is the diameter of the largest spherical cavity in the pore, d_2 is the diameter of the largest sphere which can travel through the pore and V is the pore volume shown in $Å^3$ and as percentage of the unit cell volume. Note that the ZnCar•H_2O structure has two different pores which alternate in the structure as shown in Fig. 2 of the main text. Void volumes were calculated with probe radius r=1.2 Å and grid spacing 0.1 Å.

	d_1, Å	d_2, Å	V, $Å^3$	V, %
**ZnCar•DMF**	5.18	3.78	274.3	36.0
**ZnCar**	4.58	4.12	248.4	34.2
**ZnCar•MeOH**	4.56	3.78	223.9	31.4
**ZnCar•H_2O 1**	4.36	3.58	257.0	18.0
**ZnCar•H_2O 2**	3.78	3.18	185.6	13.0

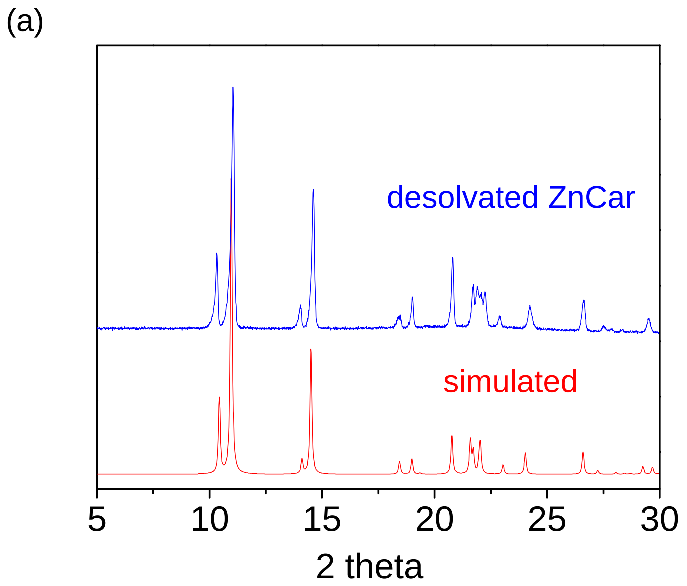

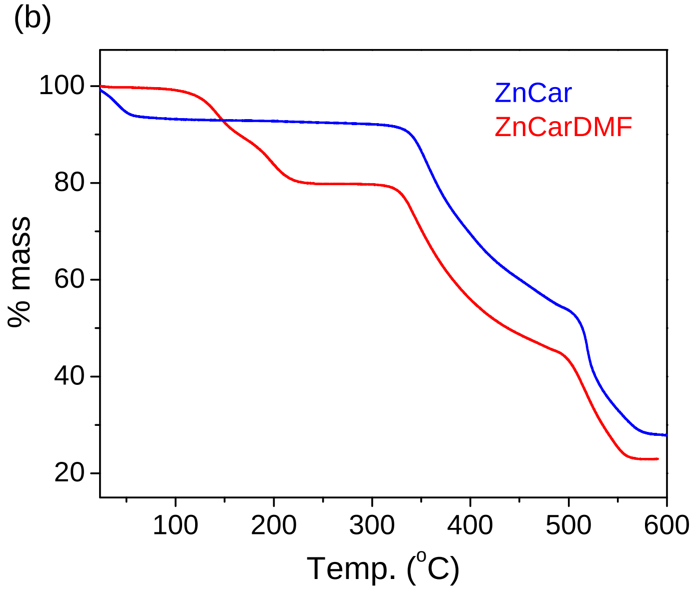

Figure S8 (a) Powder XRD pattern of desolvated ZnCar compared to simulated pattern of single crystal analysis data. (b) Thermogravimetric analysis of ZnCar (blue) under air, compared to fresh ZnCar•DMF (red), show no mass loss in the range 100 to 250°C confirming the complete removal of DMF. The mass loss, 6 % wt., up to 70°C is attributed to adsorbed H_2O on the external surface after the desolvation.

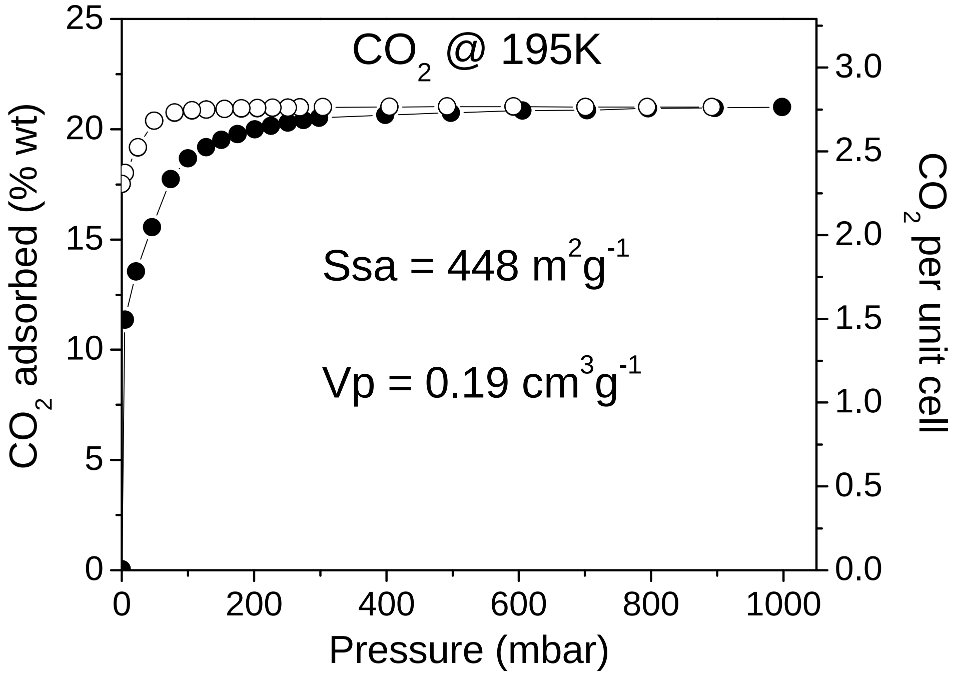

Figure S9 CO_2 adsorption desorption isotherm of ZnCar at 195 K. The adsorbed amount is expressed as % wt on the left axis and as molecules per unit cell on the right axis.

Table S4 Zn concentration of supernant solution from water stability tests after 24 h, 48 and 72 h. The Zn leaching is calculated on the basis of the total Zn added to water in the form of ZnCar•DMF.

	**Zn solution** (ppm)	**Zn leaching** (% wt)
**24h**	4.79	1.11
**48h**	5.76	1.33
**72h**	7.51	1.74

(a) (b)

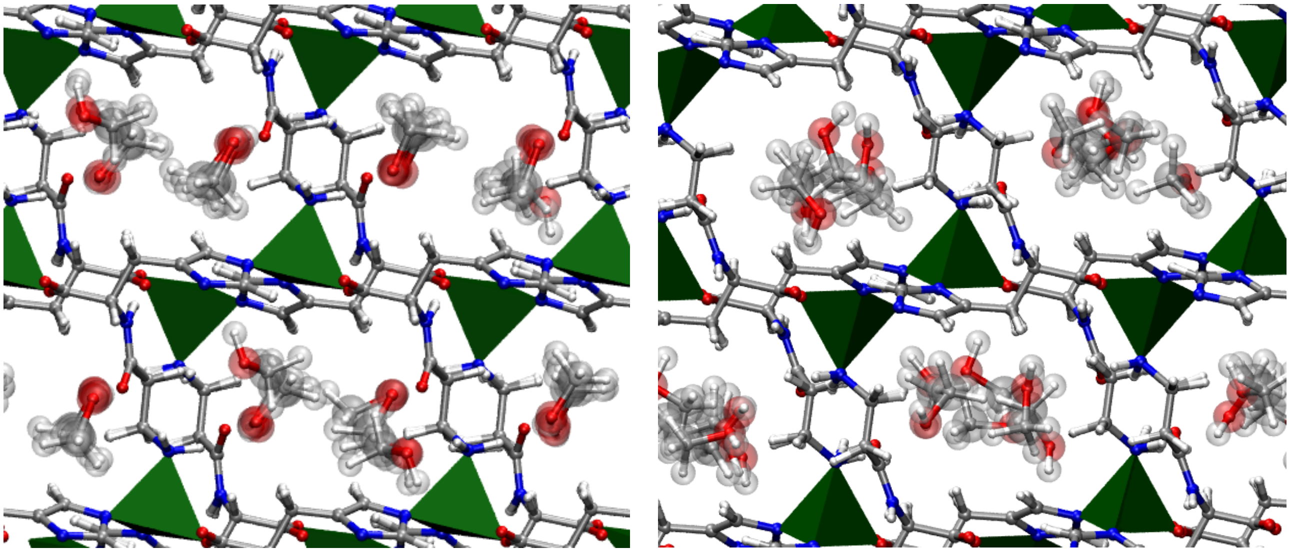

Figure S10. Snapshots of two Zn-Carnosine structures from MD simulations at 300K filled with methanol guests and viewed along the pore so several methanol molecules can be seen on top of each other. The framework in (a) has the “native” ZnCar•MeOH structure while the framework in (b) has the structure observed for the desolvated ZnCar. The coordinates of C, N, O and Zn for both frameworks were taken from the XRD and they remained fixed during the simulations. The positions of the methanol molecules were not taken from the experimental data but generated at random for both structures and then equilibrated. When the “native” ZnCar•MeOH structure is filled with methanol, approximately 85% of all guests adopt the configuration observed in the experiment – they form a double hydrogen bond with the carboxyl oxygen and the amine group. In the desolvated ZnCar structure (b), the carboxy oxygen is not facing the pore which means it cannot form an H-bond with the guest due to the excluded volume restrictions. This results in the disordered structure of methanol molecules in the pore with most guests forming hydrogen bonds with other guests.

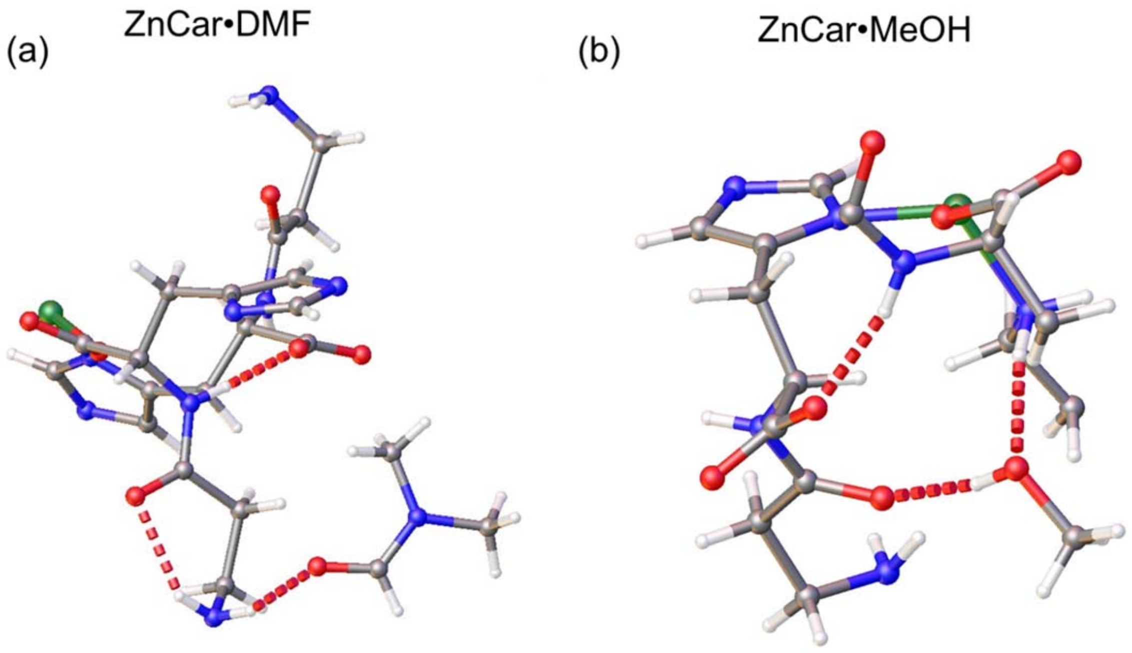

Figure S11 (a) H-bonds in the experimental collected structure of ZnCar•DMF. An intraframework between amido hydrogen and carboxylate oxygen of neihbouring carnosine, an intramolecular between carboxyl oxygen and amino hydrogen and one between DMF oxygen and amino hydrogen. (b) H-bonds in the experimental collected structure of ZnCar•MeOH. An intraframework between amido hydrogen and carboxylate oxygen of neihbouring carnosine, an H-bond between MeOH oxygen and amino hydrogen and one between carboxyl oxygen and MeOH hydrogen.

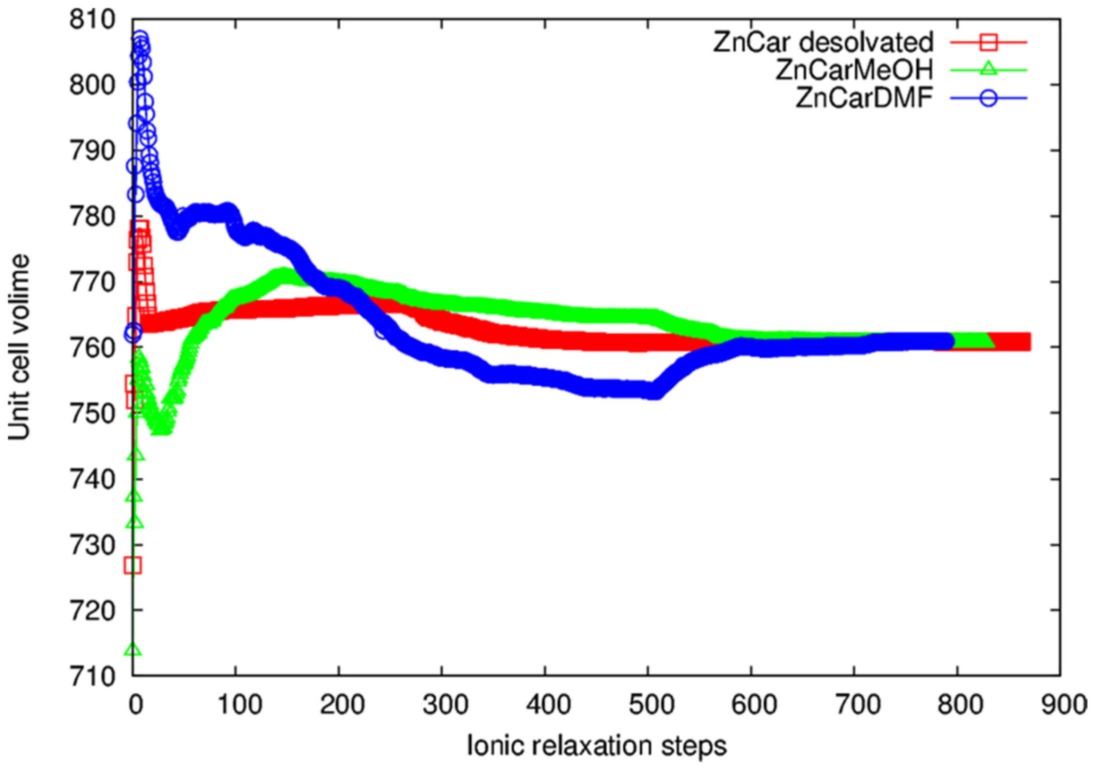

Figure S12. The evolution of the unit cell volume of three solvent-free structures during energy minimization with DFT. The initial structures were taken respectively from the XRD data of the desolvated ZnCar, ZnCar•MeOH and ZnCar•DMF unit cells. The solvent molecules were removed and the unit cell dimensions were allowed to change independently during energy minimization. All three calculations converged to the same equilibrium structure as indicated by the final energies and unit cell parameters listed in the table below.

Table S5. Unit cell parameters of DFT-minimized configurations starting from 3 experimental structures

	a, Å	b, Å	c, Å	*α*, °	*β*, °	*γ*,°	V, Å^3	E, eV
**desolvated ZnCar**	9.493	9.395	9.753	90.01	118.99	90.02	760.88	-365.7805
**ZnCar•MeOH**	9.494	9.393	9.753	90.01	119.00	90.03	760.72	-365.7806
**ZnCar•DMF**	9.489	9.394	9.753	90.00	118.98	90.02	760.50	-365.7809

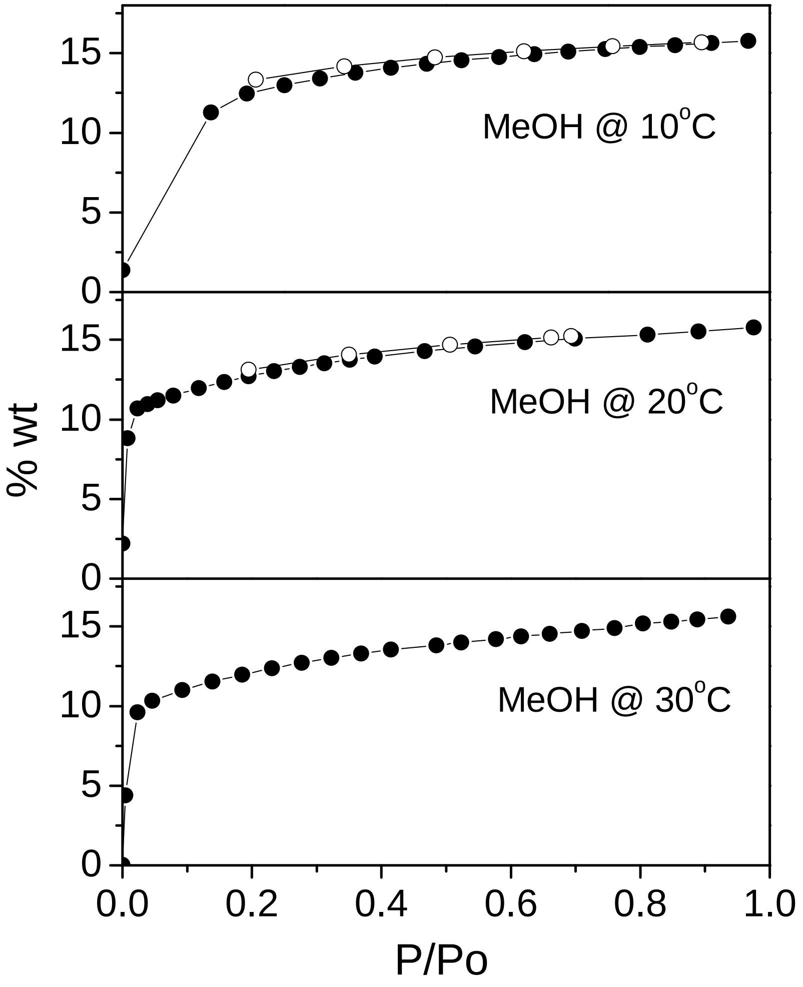

Figure S13 MeOH vapor adsorption desorption isotherms of ZnCar at 10, 20 and 30°C. The isotherms were collected on the same sample what shows that ZnCar can be loaded and unloaded with MeOH repeatedly

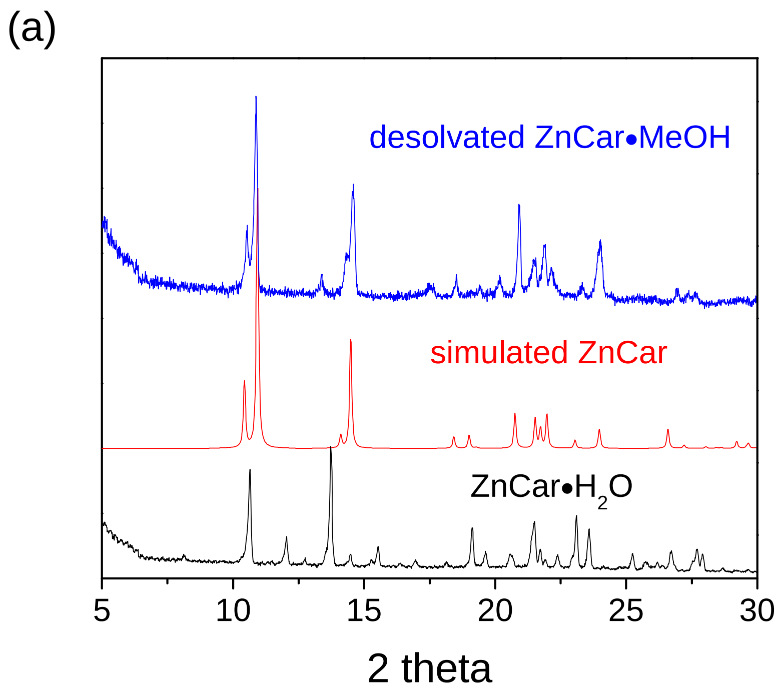

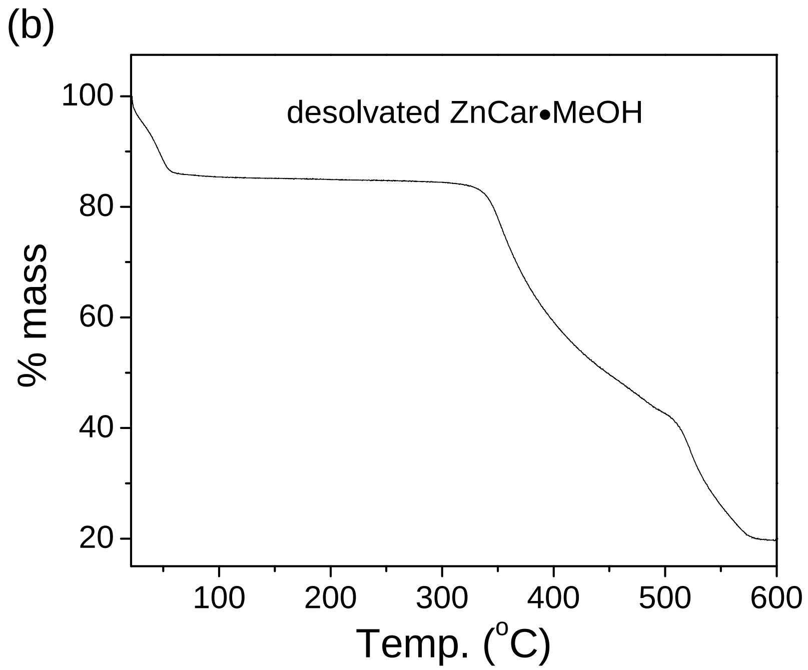

Figure S14 (a) Powder XRD pattern of desolvated ZnCar•MeOH compared to simulated pattern of desolvated ZnCar structure. There are few extra peaks of low intensity that are attributed to hydrated ZnCar from atmospheric humidity. The experimental collected pattern of ZnCar•H_2O is given. (b) Thermogravimetric analysis of desolvated ZnCar•MeOH under air. The mass loss, 14 % wt., up to 70°C is attributed to adsorbed H_2O on the external surface after the desolvation.

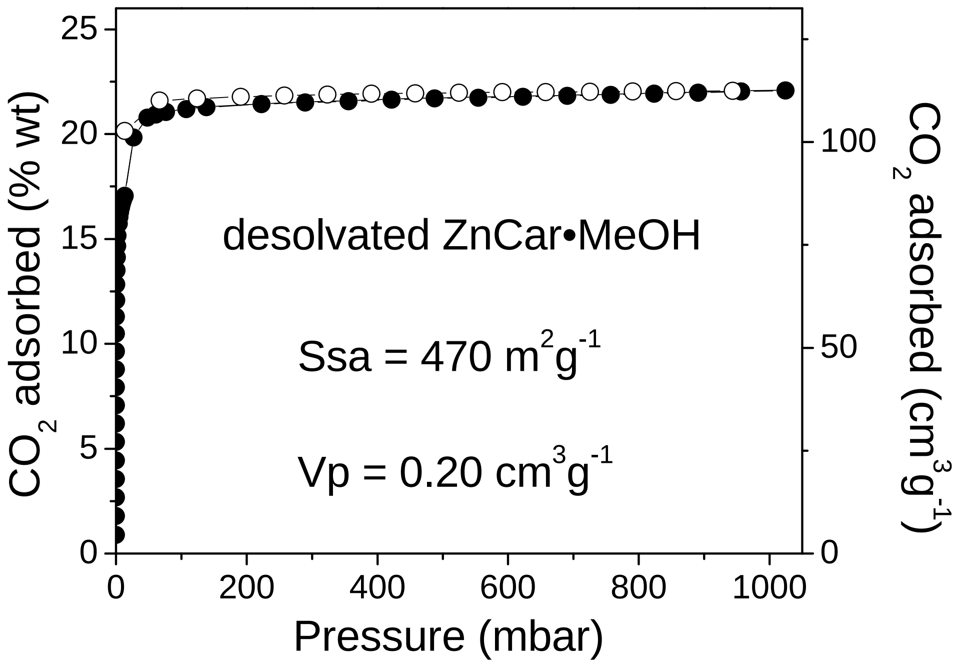

Figure S15 CO_2 adsorption desorption isotherm of desolvated ZnCar•MeOH at 195 K. The adsorbed amount is expressed as % wt on the left axis and as cm^3g^{-1} on the right axis.

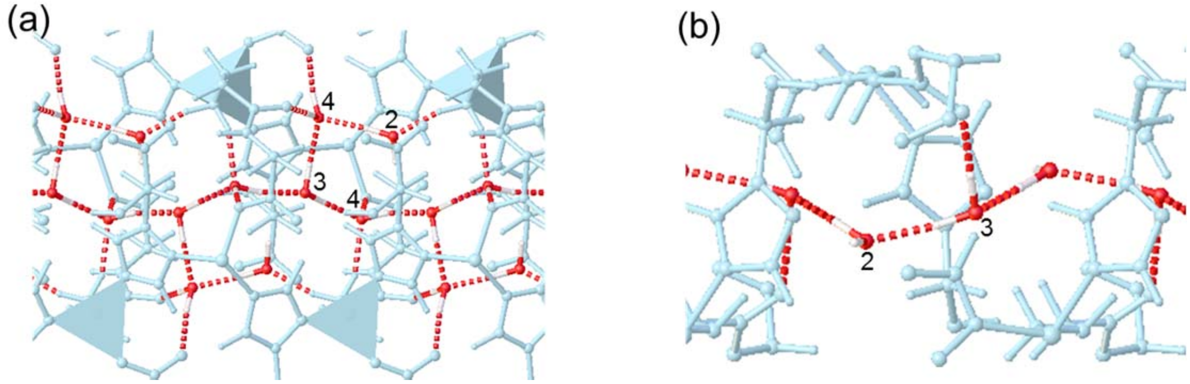

Figure S16 (a) H_2O network in the wide pore. There are 4 distinct coordinations for H_2O forming respectively 4, 4, 3 and 2 H-bonds per molecule. (b) H_2O network in the narrow pore. There are 2 distinct coordinations for H_2O forming respectively 3 and 2 H-bonds per molecule

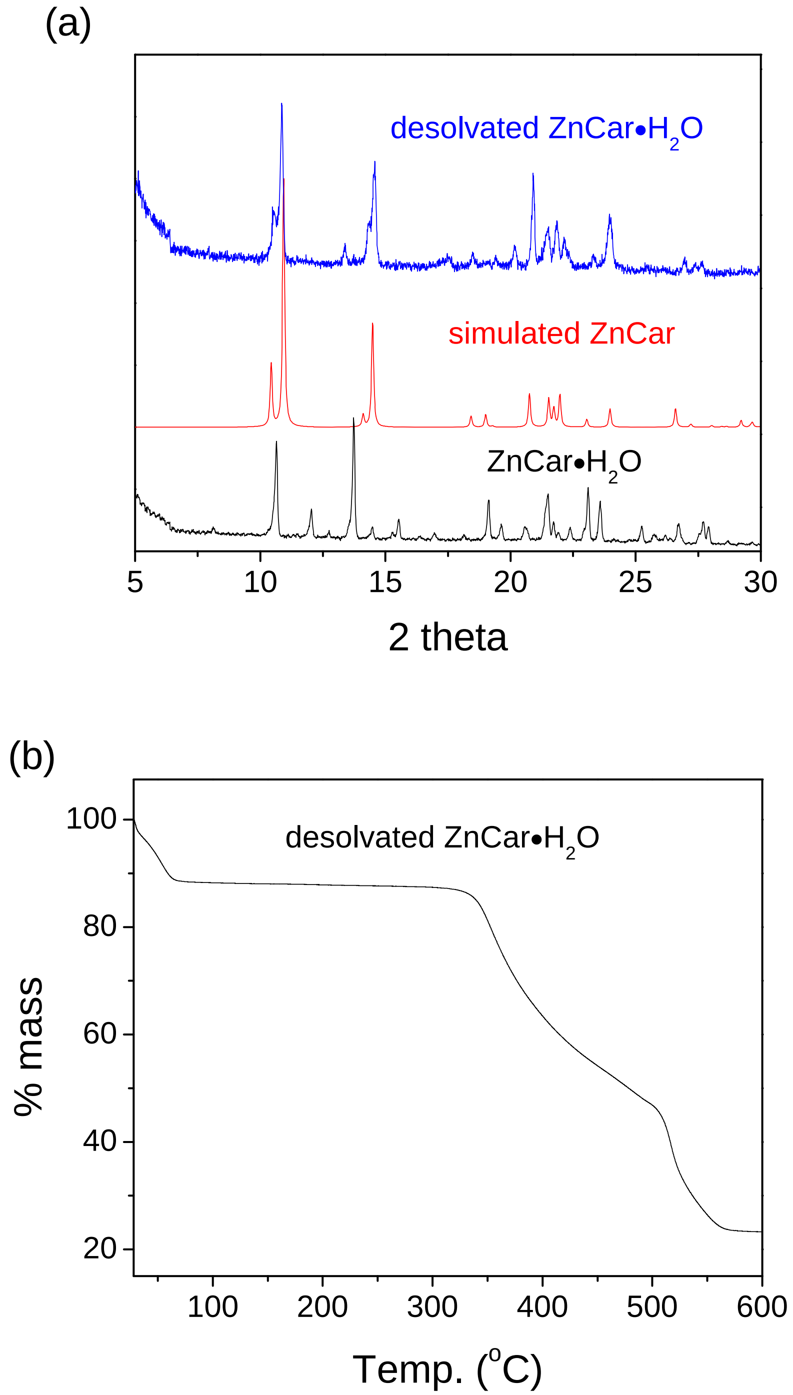

Figure S17 (a) Powder XRD pattern of desolvated ZnCar•H_2O compared to simulated pattern of desolvated ZnCar structure. There are few extra peaks of low intensity that are attributed to hydrated ZnCar from atmospheric humidity. The experimental collected pattern of ZnCar•H_2O is given. (b) Thermogravimetric analysis of desolvated ZnCar•H_2O under air. The mass loss, 11 % wt., up to 70°C is attributed to adsorbed H_2O on the external surface after the desolvation.

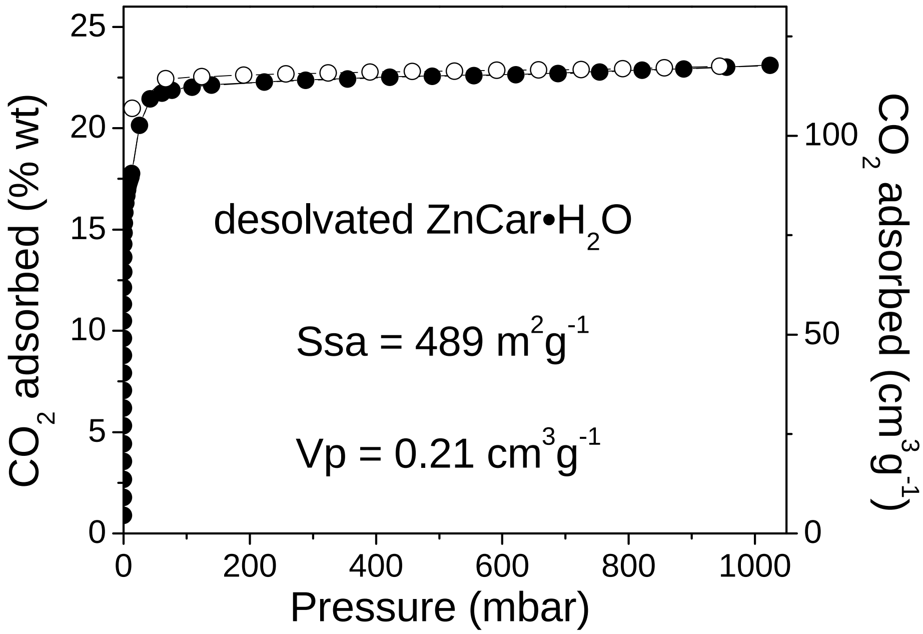

Figure S18 CO_2 adsorption desorption isotherm of desolvated ZnCar•H_2O at 195 K. The adsorbed amount is expressed as % wt on the left axis and as cm^3g^{-1} on the right axis.

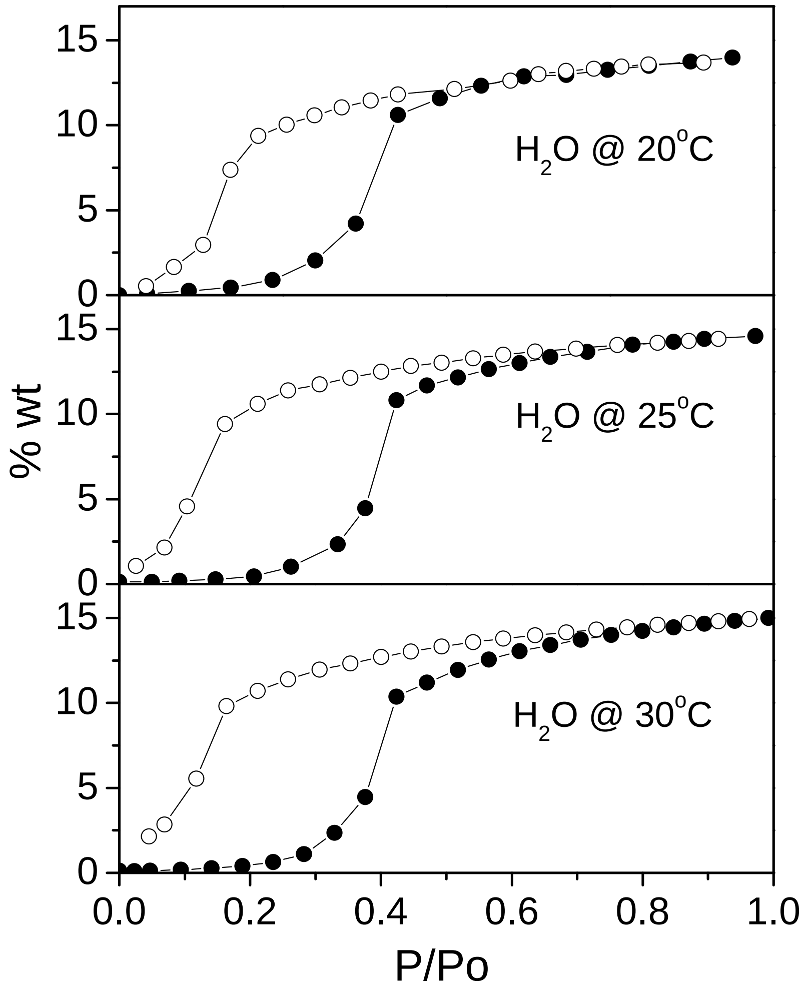

Figure S19 H_2O vapor adsorption desorption isotherms of ZnCar at 20, 25 and 30°C. The isotherms were collected on the same sample what shows that ZnCar can be loaded and unloaded with H_2O repeatedly

Table S6. DFT results for ZnCar•H_2O (no VDW correction).

	a, Å	b, Å	c, Å	α, ^{o}	β, ^{o}	γ,^{o}	V, Å^3	E, eV	ΔE, kJ/mol
**Experiment**	10.933	8.994	14.618	90.00	96.63	90.00	1,427.79		
**empty cell**	10.409	9.340	15.728	90.00	93.98	89.99	1,525.41	-731.0755	52.5
**filled cell**	11.097	9.070	14.845	90.00	96.13	90.00	1,485.71	-908.9253	

Table S7 Characteristic angles and distances within the framework, from the experimental collected crystal structures, that are weakly affected by the presence of the guest molecules:

	Zn-im-Zn angle, °	Zn-Zn distance for zincs bound to the same imidazole, Å	Zn-Zn pitch, Å	Distance between Zn-im chains, Å
**ZnCar•DMF**	138.40	5.924	9.635	9.253
**ZnCar**	139.35	5.918	9.636	9.330
**ZnCar•MeOH**	138.45	5.897	9.590	9.256
**ZnCar•H_2O 1**	133.71	5.803	9.620*	8.994
**ZnCar•H_2O 2**	136.65	5.887		

*A half of the periodic distance is given for the water cell for ease of comparison with other systems

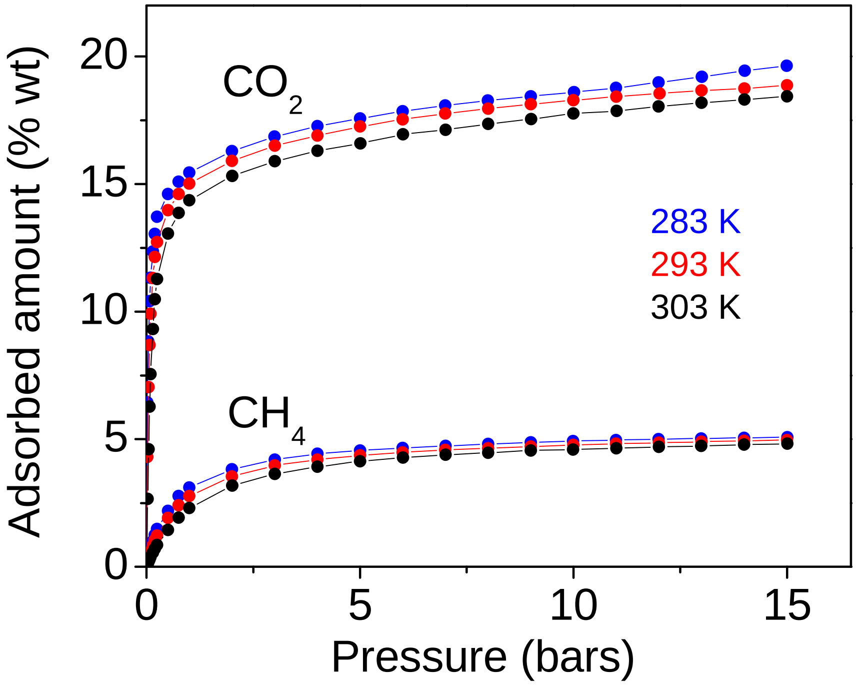

Figure S20 High pressure CO_2 and CH_4 adsorption isotherms of ZnCar at 283 K, 293 K and 303 K.

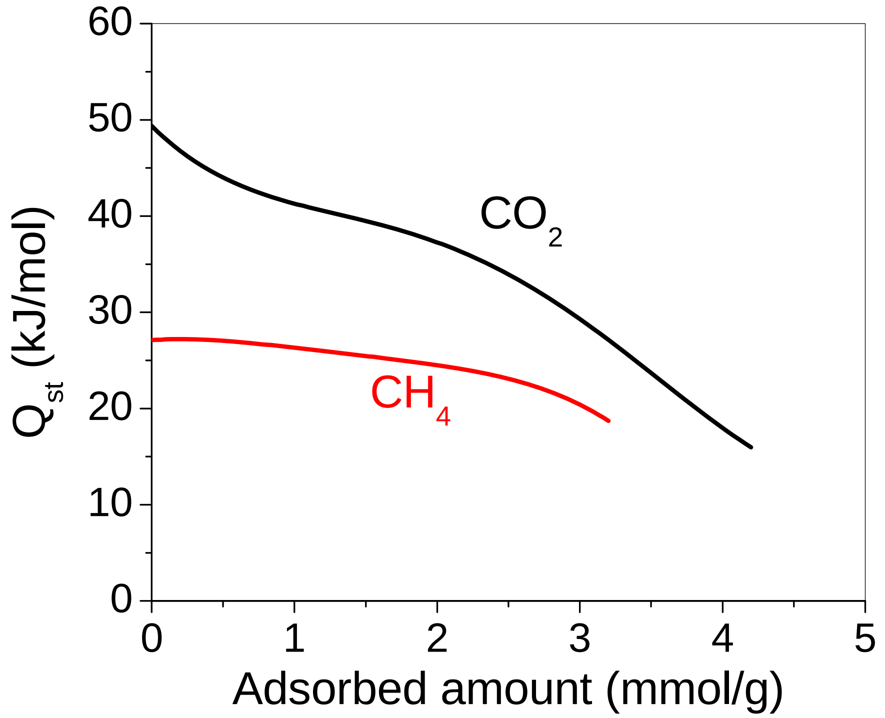

Figure S21 Isosteric heat of CO_2 and CH_4 adsorption on ZnCar

Adsorption enthalpies calculated with DFT. The unit cell parameters and the corresponding energies are shown in Table S8 after energy minimization without and with van der Waals correction. In all cases two guest molecules were placed at the positions predicted by the MD calculations (see Fig. S11 c) and then the cell geometry was allowed to relax. The adsorption energy per guest molecule was calculated as

$$\Delta E = \left(E_{\text{cell with 2 guests}} - E_{\text{empty cell}} - 2E_{\text{guest}}\right)/2$$

where E_{guest} was calculated for a single guest molecule in a gas phase. Since a plane wave basis sets method assumes the use of periodic boundary conditions, a relatively large cubic box containing a single molecule was used to represent the gas phase. We found that the box size of 15 Å was large enough to ensure that the interactions with the periodic images were below 10^{-6} eV.

Table S8 Cell parameters without and with VDW correction

Cell parameters **without** the VDW correction									
	a, Å	b, Å	c, Å	α, °	β, °	γ,°	V, Å^3	E, eV	ΔE, kJ/mol
**Experiment**	9.198	9.330	9.636	90.00	118.48	90.00	726.60		
**empty cell**	9.356	9.374	9.730	89.99	118.59	90.01	749.26	-365.8089	
**+ 2 CO_2**	9.330	9.427	9.723	89.87	118.91	90.44	748.47	-412.0044	-13
**+ 2 N_2**	9.336	9.408	9.722	90.00	118.78	90.02	748.38	-399.2188	-6
**+ 2 CH_4**	9.621	9.381	9.759	89.99	119.56	90.00	766.15	-413.9632	-3
Cell parameters **with** the VDW correction									
	a, Å	b, Å	c, Å	α, °	β, °	γ,°	V, Å^3	E, eV	ΔE, kJ/mol
**Experiment**	9.198	9.330	9.636	90.00	118.48	90.00	726.60		
**empty cell**	8.727	9.389	9.757	89.99	115.54	90.04	721.40	-366.5784	
**+ 2 CO_2**	8.953	9.149	9.732	89.88	116.92	90.37	710.75	-412.7847	-58
**+ 2 N_2**	8.633	9.286	9.725	90.01	114.99	89.88	706.54	-401.2717	-40
**+ 2 CH_4**	8.746	9.380	9.744	89.99	115.08	90.05	724.05	-417.6618	-42

Table S9 The energy values in eV for guest molecules in the gas phase

	without VDW	with VDW
**single CO_2 molecule**	-22.5022	-22.9600
**single N_2 molecule**	-16.6411	-16.9353
**single CH_4 molecule**	-24.0409	-25.1044

(a) (b)

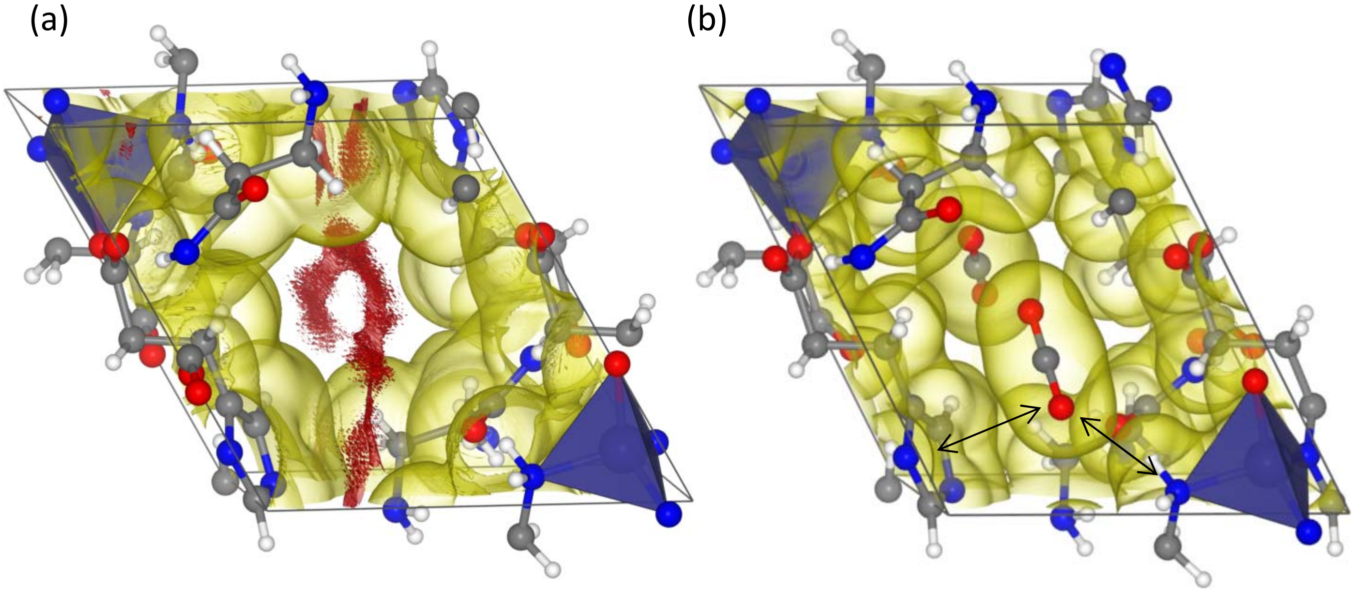

Figure S22 Equipotential surfaces calculated by DFT are shown for an empty unit cell of ZnCar (a) and for a unit cell containing two CO_2 molecules (b). The areas highlighted in red in (a) correspond to high positive electrostatic potential indicative of adsorption sites. Each of the two CO_2 molecules in (b) resides between the imidazole ring and the amine group. In this position, the oxygen on the CO_2 molecule is approximately 3.5 Å away from either the center of the imidazole ring or the nitrogen in the amine group as indicated by the black arrows in (b).

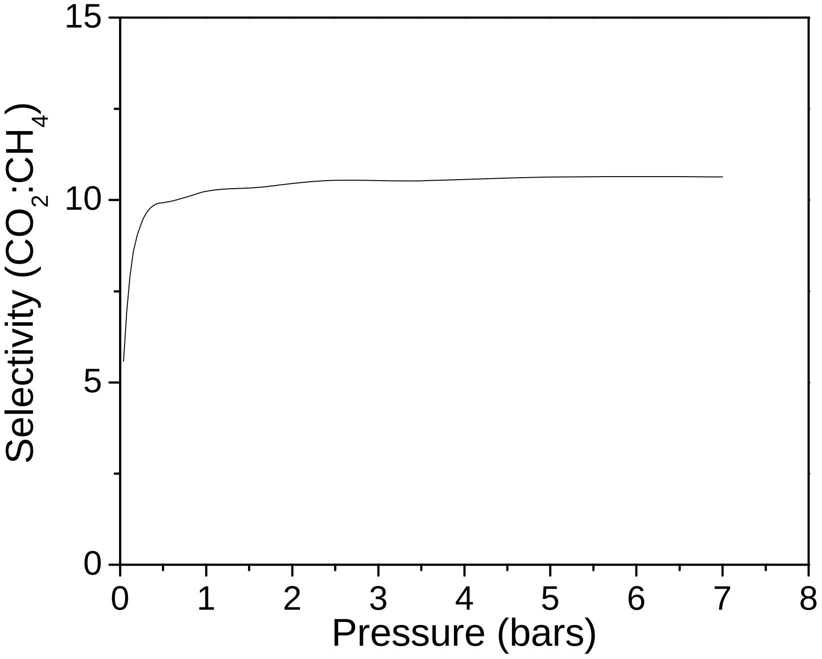

Figure S23 Adsorption selectivity of CO_2 over CH_4 on ZnCar at 303 K estimated with IAST.

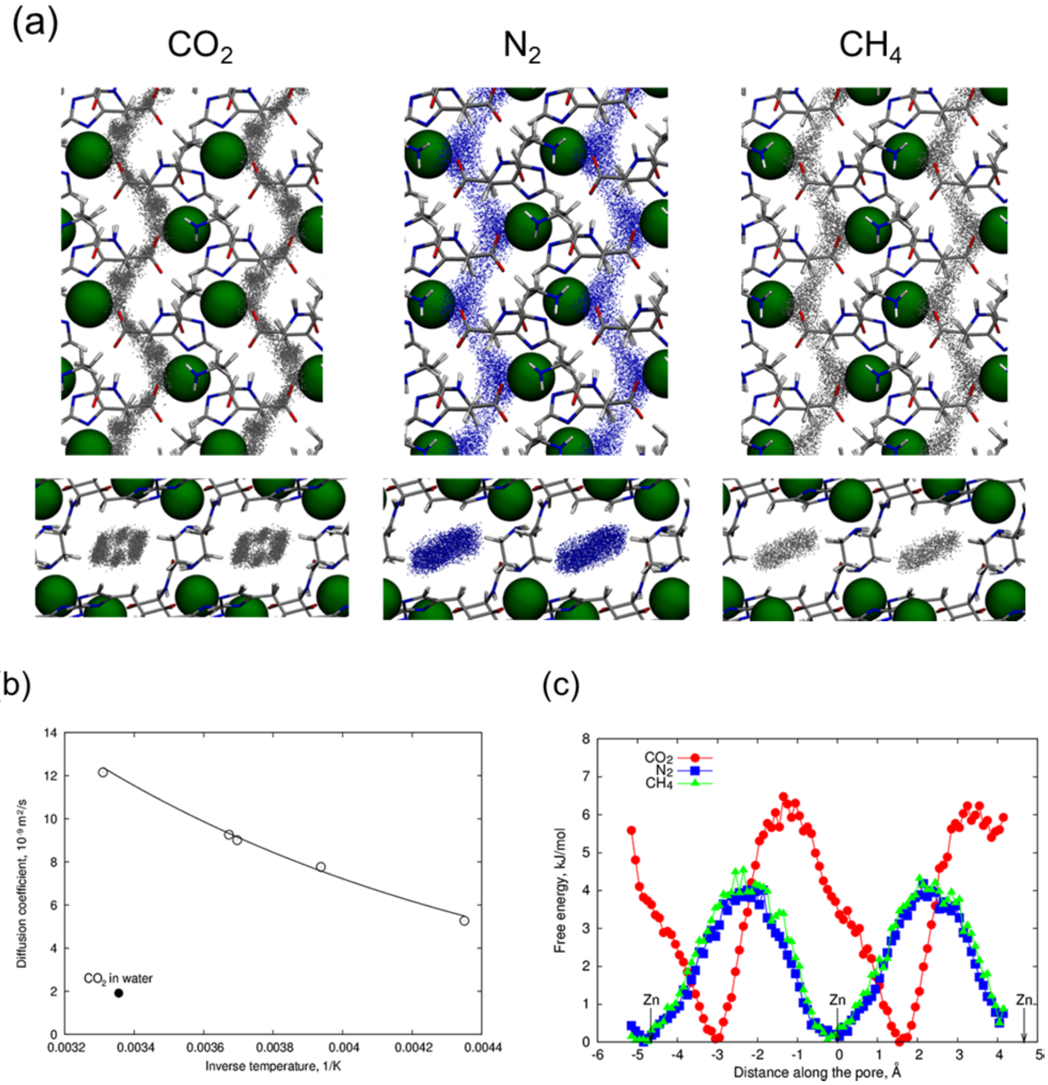

Figure S24 (a) Positions of guest molecules calculated in 15 ns MD runs at 298 K are shown for CO_2, N_2 and CH_4 parallel and perpendicular to the direction of the pore. (b) CO_2 diffusion coefficient calculated at different temperatures and fitted using an Arrhenius expression $D = D_0 e^{-\frac{E}{kT}}$ to give an activation energy of E=6.5 kJ/mol. (c) The free energy profiles calculated from the probability densities depictured in (a) for a single unit cell. While both N_2 and CH_4 reside near the zinc cation, the equilibrium position of CO_2 is offset by about 1.7 Å. At 300 K, the diffusion coefficients for CO_2 is about 12.5×10^{-9} m^2/s, while for CH_4 and N_2 it is respectively 2 and 1.5 times faster. The activation barrier for both CH_4 and N_2 is close to E=4.1 kJ/mol which means that their temperature dependence is weaker than that of CO_2. Thus at 195 K, the diffusion

coefficients for CO_2 is about 3.0×10^{-9} m^2/s, while for CH_4 and N_2 it is respectively 3.3 and 2.5 times faster.

Theoretical estimates for guest residence time associated with the diffusion inside and diffusion out of the ZnCar framework. The diffusion coefficients calculated in SIX can be related to guest resident rime inside the large cavity (i.e. the time between the "hops") and can also be directly compared to the residence time at the framework interface (i.e. the time required to leave the surface and go to the gas phase). For the one-dimensional diffusion we can express the mean-square displacement of a guest molecule along the z direction either via the Einstein's equation for Brownian motion:

$$< \boldsymbol{z^2(t)} >= \boldsymbol{2Dt}$$

or as a displacement of a random walk with step *l*:

$$< \boldsymbol{z^2(t)} >= \boldsymbol{l^2N(t)}$$

where $N(t) = t/\tau$ is the number of steps in the random walk made over a long period of time *t* and τ is the residence time. Combining the above equations and using Arrhenius expression $D = D_0 e^{-\frac{E}{kT}}$, we arrive at the estimate for the residence time:

$$\tau = \frac{l^2}{2D_0} e^{\frac{E}{kT}}$$

where l=4.82Å is a half of the unit cell parameter *c* and energy E=6.5 kJ/mol for CO_2 diffusion. This equation can also be used to estimate the residence time at the interface τ_{int} if we use the isosteric heat of adsorption of 49 kJ/mol for CO_2 as the activation barrier E. The table below shows calculated residence times for both CO_2 and CH_4 at 195 K and 298 K.

Table S10. Estimated residence time for diffusion in bulk and across the interface

		CO_2	CH_4
Bulk diffusion, τ	195 K	38 ps	12 ps
	298 K	10 ps	5 ps
Interface hopping, τ_{int}	195 K	950 μs	21 μs
	298 K	0.7 μs	0.06 μs

Table S10 shows that the diffusion rate inside the framework is 4 to 7 orders of magnitude faster than the rate at which guests leave the sample. Assuming the loading of 2 guests per unit cell and instantaneous diffusion through a sample of L=1mm in length, we can estimate the time required for all guests to vacate the sample as $T = \frac{L}{l}\tau$.

Table S11. Estimated time required to empty a 1mm-long sample

		CO_2	CH_4
Evacuation time, T	195 K	33 min	45 s
	298 K	1.4 s	0.1 s

Table S11 shows that for CO_2 at 195 K the residence time at the interface is the rate limiting process in evacuating CO_2 consistent with the experimental observations.

REFERENCES

1. J. Cosier and A. M. Glazer, *J. App.Cryst.,* **1986**, *19*, *105-107.*
2. SAINT v7.68A (Bruker, 2009)
3. SADABS-2008/4 (Bruker,2010)
4. SHELXS and SHELXL, G.M. Sheldrick, *Acta Cryst.,* **2008**, *A64*, 112-122
5. O. V. Dolomanov, L. J. Bourhis, R. J. Gildea, J. A. K. Howard and H. Puschmann, OLEX2: a complete structure solution, refinement and analysis program. *J. Appl. Cryst.,* **2009**, *42*, 339-341
6. CCVT Rigaku, 2011; C. Marti-Gastaldo, J. E. Warren, K. C. Stylianou, N. L. O. Flack, and M. J. Rosseinsky, *Angewandte Chemie, (International ed. in English)*, 2012, **51**, 11044–8
7. J. E. Warren, STANAL: Statistics and Analysis - data extraction and compilation software, developed for analysis large data set strucutres. (**2011**)
8. J. E. Warren, CCVT2LOG: - CCVT log parsing and analysis tool - data extraction and compilation software, developed for analysis large CCVT data set strucutres. (**2011**)
9. J. E. Warren, FMD: Free My Data - an automated frame conversion program utilising Simon Parson's Eclipse and Bruker Frm2Frm executables. (**2010**)
10. APEX2 (Bruker AXS Inc. **2009**)
11. L.J. Bourhis, O.V. Dolomanov, R.J. Gildea, J.A.K. Howard, H. Puschmann, in preparation, **2012**
12. Fung, B. M.; Khitrin, A. K.; Ermolaev, K. *J. Magn. Reson.* **2000**, *142*
13. Morcombe, C. R.; Zilm, K. W. *J. Magn. Reson.* **2003**, *162*, 479
14. Rouquerol, J.; Llewellyn, P.; Rouquerol, F. *Stud. Surf. Sci. Catal.* **2007**, *160*, 49
15. Czepirski, L.; Jagiello, J. *Chem. Eng. Sci.* **1989**, *44*, 797–801
16. Myers, A. L. *Adsorption* **2003**, *9*, 9
17. G. Kresse and J. Hafner, *Phys. Rev. B*, *47*, 558, (1993).
18. P. E. Blochl. Projector augmented-wave method. *Phys. Rev. B*, **50**, 17953, (1994).
19. J. P. Perdew, K. Burke, and M. Ernzerhof, *Phys. Rev. Lett.*, **77**, 3865, (1996).
20. M. Dion, H. Rydberg, E. Schröder, D. C. Langreth, and B. I. Lundqvist, *Phys. Rev. Lett*. **92**, 246401 (2004)
21. Phillips et al., *J. Comp. Chem.,* **26**, 1781-1802 (2005)
22. Pang, Y.-P., *Proteins*, **45**, 183-189, (2001)
